# Epigenetic changes in histone acetylation underpin resistance to the topoisomerase I inhibitor irinotecan

**DOI:** 10.1093/nar/gkw1026

**Published:** 2016-10-26

**Authors:** Cornelia Meisenberg, Mohamed E. Ashour, Lamia El-Shafie, Chunyan Liao, Adam Hodgson, Alice Pilborough, Syed A. Khurram, Jessica A. Downs, Simon E. Ward, Sherif F. El-Khamisy

**Affiliations:** 1 Mammalian Genome Stability Group, Krebs and Sheffield Institute for Nucleic Acids, University of Sheffield, Western Bank, Sheffield S10 2TN, UK; 2 The Wellcome Trust DNA Repair Group, University of Sussex, Brighton BN1 9RQ, UK; 3 Center for Genomics, Helmy Institute for Medical Sciences, Zewail City for Science and Technology, Giza, Egypt; 4 Unit of Oral and Maxillofacial Pathology, School of Clinical Dentistry, University of Sheffield, UK; 5 Genome Damage and Stability Centre, University of Sussex, Brighton BN1 9RQ, UK; 6 Sussex Drug Discovery Centre, School of Life Sciences, University of Sussex, Brighton BN1 9QJ, UK

## Abstract

The topoisomerase I (TOP1) inhibitor irinotecan triggers cell death by trapping TOP1 on DNA, generating cytotoxic protein-linked DNA breaks (PDBs). Despite its wide application in a variety of solid tumors, the mechanisms of cancer cell resistance to irinotecan remains poorly understood. Here, we generated colorectal cancer (CRC) cell models for irinotecan resistance and report that resistance is neither due to downregulation of the main cellular target of irinotecan TOP1 nor upregulation of the key TOP1 PDB repair factor TDP1. Instead, the faster repair of PDBs underlies resistance, which is associated with perturbed histone H4K16 acetylation. Subsequent treatment of irinotecan-resistant, but not parental, CRC cells with histone deacetylase (HDAC) inhibitors can effectively overcome resistance. Immunohistochemical analyses of CRC tissues further corroborate the importance of histone H4K16 acetylation in CRC. Finally, the resistant clones exhibit cross-resistance with oxaliplatin but not with ionising radiation or 5-fluoruracil, suggesting that the latter two could be employed following loss of irinotecan response. These findings identify perturbed chromatin acetylation in irinotecan resistance and establish HDAC inhibitors as potential therapeutic means to overcome resistance.

## INTRODUCTION

Irinotecan is converted into its active form SN-38, which is a camptothecin (CPT)-based agent that promotes cancer cell death by interfering with the topoisomerase type 1β enzyme (TOP1) (1). TOP1 is involved in DNA relaxation to promote cellular activities such as transcription and DNA replication (2,3). Whilst DNA cleavage and re-ligation by TOP1 is a fast process, TOP1 poisons prevent the re-ligation of reversible TOP1 cleavage complexes (TOP1cc), resulting in covalently trapped TOP1 protein-linked DNA breaks (PDBs) (3–6). PDBs are irreversible and removal of TOP1 by proteasomal degradation is required for subsequent repair. Upon TOP1 degradation, tyrosyl DNA phosphodiesterase 1 (TDP1) processes the remaining 3΄-phospho-tyrosyl peptide in a PARP1-dependent manner prior to repair completion by the DNA single-strand break repair pathway (SSBR) (7–12). Indeed, the majority of TOP1-PDBs are repaired in this way (13–15). If an advancing replication fork encounters a TOP1cc or an unrepaired TOP1-PDB on the leading strand, the forks are reversed and stabilised by PARP1 to allow time for the removal of TOP1-PDBs (16), a process that is negatively regulated through the RecQ1 helicase (17,18). Failure to repair TOP1-PDBs at replication forks ultimately results in replication run-off and the generation of a DNA double-strand break (DSB) (19,20). DNA DSBs trigger the DNA damage response, including cell cycle arrest mediated by both ATM and ATR, γH2AX signalling and p53-regulated apoptosis (6,21,22). TOP1 can also be removed from TOP1-PDBs by nucleolytic cleavage of DNA, removing TOP1 and a stretch of DNA to which it is attached. This is conducted by a number of nucleases including the Mus81-Eme1 heterodimer bound to the scaffold protein SLX4 that additionally carries SLX1 (23–25). The XPF-ERCC1 endonuclease is also implicated in TOP1 removal in an SLX4 independent manner (24,26). Once excised, the remaining DSB is repaired through homologous recombination (HR)-mediated DSB repair involving both the DNA damage response complex MRN and the end processing factor CtIP (27–29). Persistence of unrepaired PDBs and the generation of DSBs underlie the clinical utility of TOP1 poisons as anti-cancer drugs.

Despite their broad application in the clinic, resistance to TOP1 poisons remains an unmet clinical challenge. Recent studies have focused on identifying molecular biomarkers for predicting irinotecan sensitivity (30,31). Classical mechanisms for loss of sensitivity such as loss of drug conversion to its active metabolite or gain of drug pump functions have been reported (32,33). Inhibition of the ABCG2 drug efflux pump using sorafenib was shown to sensitise both non-resistant and irinotecan resistant CRC cells to irinotecan (34). The inability to trigger cell cycle arrest (G2/M arrest) and p53-mediated apoptosis in response to CPT can also promote loss of CPT sensitivity (35). TOP1 downregulation and inactivating mutations that reduce the trapping of TOP1 on DNA have also been reported as possible mechanisms of CPT resistance (35,36). Finally, hyperactivity of factors of the aforementioned SSBR and HR DNA repair pathways may also account for resistance onset to TOP1 poisons. For example, upregulation in the level or activity of TDP1, CtIP, XPF-ERCC1 and Mus81-Eme1 is known to protect cells from CPT-mediated damage (35,37–39). Although much is known about changes in *bona fide* DNA repair factors as modulators of CPT response, little is known about the role of epigenetics, particularly chromatin acetylation in this process.

Here, we generated CRC models of irinotecan (CPT-11) resistance derived from two independent cell lines to investigate the mechanism of resistance onset, cross-resistance with other CRC targeting therapies and novel means by which to overcome resistance. Our findings reveal that irinotecan resistance is neither due to modulation of the main cellular target of irinotecan, TOP1, nor upregulation of the key TOP1 repair factor, TDP1. Instead, we reveal that the faster repair of PDBs and the improved ability to re-start irinotecan-arrested forks are the driver of resistance. Changes in *bona fide* PDB repair proteins are not driving resistance but instead perturbations in histone H4K16 acetylation and rate of 53BP1 recruitment to damage sites is the underlying mechanism of resistance. Consequently, histone deacetylase inhibitors can mechanistically reverse resistance. Finally, we identify cross-resistance with oxaliplatin but not with ionising radiation or 5-fluoruracil, suggesting that the latter two could be employed following loss of irinotecan response.

## MATERIALS AND METHODS

### Cells and reagents

The human colorectal cancer cell lines RKO and DLD1 (ATCC, LGC Standards, Middlesex, UK) and their CPT-11 resistant derivative single clones were maintained in a 5% CO_2_ incubator at 37°C and grown in Gibco RPMI media (Life Technologies, Paisley, UK) supplemented with 10% FCS and 2 mM l-glutamine. Irinotecan hydrochloride (CPT-11), 5-fluorouracil (5-FU), oxaliplatin, hydroxyurea (HU), camptothecin (CPT), doxorubicin, etoposide and SN-38 were obtained from Sigma-Aldrich (Gillingham, UK) whilst the PARP inhibitor, olaparib (AZD2281) was obtained from Stratech Scientific Limited (Suffolk, UK). Trichostatin A (TSA) and Panobinostat (LBH-589) were obtained from Selleckchem.

### Generation of irinotecan resistant clones

RKO and DLD1 cells were maintained in RPMI (10% FCS, 2 mM l-glutamine) media supplemented with either a daily low dose of CPT-11 (0.1 μM, treatment culture 1), daily moderate CPT-11 dose (0.5 μM, treatment culture 2), daily CPT-11 dose that increased at every split (0.1, 0.2, 0.3 μM, etc. up till 1 μM, treatment culture 3) or a high CPT-11 dose (1 μM increasing to 2 μM, treatment culture 4) that was repeated post-recovery. Splits were carried out twice a week and recovery lasted ∼7 days whilst the treatment period spanned 2 months. Selection of single clones was carried out in a 96-well format using 2 μM CPT-11 and surviving single colonies were expanded, stored and maintained in CPT-11-free media for further analysis.

### Clonogenic survival assay

Cell sensitivity to irinotecan (CPT-11), camptothecin (CPT), oxaliplatin, 5-fluorouracil (5-FU), hydroxyurea (HU), olaparib, SN-38, doxorubicin, etoposide, trichostatin A (TSA), panobinostat (LBH-589) and irradiation was measured by clonogenic survival assay. Adhered cells seeded at dose dependent densities in 6 or 10 cm dishes were treated with irradiation or media containing drug for the duration of colony formation (9 days). Colonies were subsequently fixed and stained using 0.4% methylene blue solution in 50% methanol or 1% Giemsa in methanol and the colonies containing >50 cells were counted as surviving colonies. The surviving fraction was calculated as (colonies counted/total cells seeded)^treated^/(colonies counted/total cells seeded)^untreated^).

### mRNA silencing

mRNA silencing was carried out using Lipofectamine 2000 or 3000 RNAiMAX reagent (Invitrogen, Paisley, UK) as described previously (37). Briefly, a mixture of 10 μl Lipofectamine 2000 RNAiMAX reagent in 250 μl FCS-free media was incubated at room temperature for 5 min prior to mixing with 250 μl FCS-free media containing siRNA oligonucleotides for TDP1 (ON-TARGET*plus* smartpool, Fisher Scientific, Loughborough, UK), RECQ1 (5΄-GAAGAUUAUUGCACACUUUUUtt-3΄), PARP-1 (5΄-AGAUAGAGCGUGAAGGCGAtt-3΄, 5΄-AAGAUAGAGCGUGAAGGCGAAtt-3΄), MUS81 (5΄-CAGCCCUGGUGGAUCGAUAtt-3΄, 5΄-CAGGAGCCAUCAAGAAUAAtt-3΄), XFP/ERCC4 (5΄-CCAAACAGCUUUAUGAUUUtt-3΄, 5΄-GCACCUCGAUGUUUAUAAAtt-3΄, 5΄-CGGAAGAAAUUAAGCAUGAtt-3΄, 5΄-UGACAAGGGUACUACAUGAtt-3΄), CtIP (5΄-GCUAAAACAGGAACGAAUCUUtt-3΄), KU70/XRCC6 (5΄-UUCUCUUGGUAACUUUCCCtt-3΄), or the BLAST validated scrambled siRNA sequence (5΄-UUCUUCGAACGUGUCACGUtt-3΄) or (5΄-GCGCGCUUUGUAGGAUUCGtt-3΄). The mixture was incubated for 20 min and added dropwise onto dishes containing 3 × 10^5^ cells in 3 ml media. A second transfection was carried out 6 or 24 h later, media replaced 24 h after the first transfection and cells subjected to survival assays and qPCR analyses.

### Quantitative PCR

Cells were harvested for RNA extraction according to the manufacturer instructions (RNeasy Mini Kit, Qiagen). Reverse transcription was conducted from the respective mRNA transcripts using High-Capacity cDNA Reverse Transcription Kit (Applied Biosystems). Maxima SYBR Green 2X (Thermo Scientific) was added to the cDNA-primer mix and qPCR performed using the following primers: PARP-1 (F: 5΄-CCAACTACTGCCATACGTCTCA-3΄ ; R: TTAGCTGAAGGATCAGGGGTAG), XPF/ERCC4 (F: 5΄-GGGCATTGACATTGAACCCG-3΄; R: 5΄-CTGTAGAGGCGGCCGTTATT-3΄), CtIP (F: 5΄-GAGCACTCTGTGTGTGCAAATG-3΄; R: 5΄-GTTCCATGTG CTTTGGCCATTG-3΄), SLX4 (F: 5΄-GGAACTGGATAGGTTTGGAGTC-3΄; R: CTGCAACAGCGGCTGTGAGGAC-3΄), GAPDH (F: 5΄-TTCGTCATGGGTGTGAACCA-3΄; R: TGATGGCATGGACTGTGGTC-3΄) and KU70 (F: 5΄-TCTTGGCTGTGGTGTTCTATGGT-3΄; R: 5΄-GAGTGAGTAGTCAGATCCGTGGC-3΄). Fold changes were calculated using the following formula: Fold change = 2^−ΔΔCT^, where ΔΔC = [CT(target,untreated) −CT(GAPDH,untreated)] − [CT(target,treated) − CT(GAPDH,treated)] (40).

### Whole cell extract

Adhered cells were washed twice with ice cold PBS (phosphate buffered saline), collected by centrifugation at 1500 rpm for 5 min and extraction carried out for 30 min using ice-cold lysis buffer (20 mM Tris–HCl pH 7.5, 10 mM EDTA pH 8.0, 100 mM NaCl, 1% Triton X-100) supplemented with Complete Mini EDTA-free Protease Inhibitor Cocktail (Roche Applied Science, Burgess Hill, UK). The lysate was cleared by centrifugation at 13 000 rpm for 10 min and the supernatant collected as whole cell extract (WCE). Bradford assay was used to measure protein concentration and the samples stored at −80°C.

### Western blotting

WCE (40 μg) was separated by 10% SDS-polyacrylamide gel electrophoresis (PAGE) at 125 V for 2 h and transferred onto a Hybond-C Extra Nitrocellulose membrane (Fisher Scientific UK, Loughborough, UK) at 25 V for 90 min. The membrane was blocked in 5% PBS-milk for 1 h and probed overnight with 5% PBST-milk containing antibodies against TDP1 (ab4166; Abcam, Cambridge, UK), TOP1 (SC-32736; Santa Cruz Biotechnologies, California, USA), PARP1 and cleaved PARP1 (A6.4.12, Biorad, Hemel Hempstead, UK), pP53-pSER15 (#9284, Cell Signalling Technology, Leiden, Netherlands), p21 (SC417; Santa Cruz Biotechnologies, California, USA), Mus81 (ab14387, Abcam, Cambridge, UK), RecQ1 (H-110; sc-25547; Santa Cruz), RPA (LS-C 38952, Lifespan biosciences), BRCA2 (H-300, SC-8326, Santa Cruz), Rad51 (H-92 SC-8349, Santa Cruz), histone H4K16Ac (#61529, Active Motif, Belgium), histone H3K9Ac (ab4441, Abcam, Cambridge, UK), histone H3K56Ac (#39281, Active Motif, Belgium), histone H3K14Ac (#39599, Active Motif, Belgium) and actin (A4700; Sigma-Aldrich, Gillingham, UK), TDP2 (AP33010-P050, Aviva Systems Biology), MRP4 (ab56675, Abcam), XPF (Ab-1 (219), Rad17 (H3, Santa Cruz), pATM S1981 (Abcam), ATM (D2E2, Cell Signaling). Membranes were washed in PBST three times prior to a 1 h incubation with HRP-labelled polyclonal goat anti-rabbit or polyclonal rabbit anti-mouse secondary antibodies (Dako, Ely, UK) at a 1:3000 dilution in 5% PBST-milk. Membranes were then washed in PBST three times prior to film development using the SuperSignal West Pico Chemiluminescent Substrate (Fisher Scientific UK, Loughborough, UK) and band quantification using ImageJ software.

### TDP1 activity assay

*In vitro* 3΄-tyrosyl-DNA phosphodiesterase activity was measured as previously described (37). Briefly, 10 μl reaction volumes containing 50 nM 5΄-Cy5.5 labelled substrate (5΄-(Cy5.5)GATCTAAAAGACT(pY)-3΄) (Midland Certified Reagent Company, Texas, USA) and the indicated amounts of WCE (ng) or recombinant human TDP1 (7) in assay buffer (25 mM HEPES, pH 8.0, 130 mM KCl, 1 mM DTT) were incubated at 37°C for 1 h. Reaction products were mixed with 10 μL loading buffer (44% deionized formamide, 2.25 mM Tris-borate, 0.05 mM EDTA, 0.01% xylene cyanol, 1% bromophenol blue), heated to 90°C for 10 min and separated on a 20% Urea SequaGel (Fisher Scientific, Loughborough, UK) at 190 V for 2 h in 1× TBE. A FujiFilm Fluor Imager FLA-5100 was used to take images at 635 nm and bands quantified using ImageJ software.

### Immunostaining

Cells grown to sub-confluent densities on coverslips were treated with DMSO, CPT-11 (1, 2 or 5 μM) or 0.5 mM hydroxyurea in media at 37°C for 1.5 h. Coverslips were washed twice with PBS and recovered in drug-free media for indicated time points. Coverslips were subsequently washed twice in ice-cold PBS prior to fixing with 3.7% paraformaldehyde for 10 min and permeabilising in 0.2% Triton-X in PBS for 3 min. Coverslips were washed three times in PBS, blocked in 2% BSA-Fraction V (Sigma-Aldrich, Gillingham, UK) for 30 min and incubated with primary antibodies against γH2AX (JBW301, Millipore, Watford, UK), Rad51 (H-92, Santa Cruz Biotechnologies, California, USA), pRPA (A300-245A, Bethyl Laboratories, Texas, USA) and 53BP1 (A300-272A, Bethyl Laboratories, Texas, USA) at dilutions of 1:800, 1:300, 1:200 and 1:400 in 2% BSA-Fraction V, respectively. Coverslips were again thrice washed in PBS prior to incubation with FITC-labelled anti-mouse or Cy3-labeled anti-rabbit secondary antibodies (Sigma-Aldrich, Gillingham, UK) at a 1:300 dilution in 2% BSA-Fraction V for 45 min. Coverslips were then washed three times in PBS and mounted using DAPI Vectashield (Vector Laboratories, Peterborough, UK). Cells were visualised on a Nikon Eclipse e-400 microscope and the number of foci per cell was counted. For olaparib and TSA treatment, cells were pre-incubated with 1 μM olaparib or 1 μM TSA for 1 and 2 h, respectively, prior to further drug treatment. Recovery was carried out in media containing 1 μM olaparib or 1 μM TSA.

### Measurement of TOP1 cleavage complexes (TOP1cc): modified alkaline COMET assay

The modified alkaline COMET assay (MACA) was modified from (41) and performed as described (22) to measure TOP1-cleavage complexes (Top1-ccs). Cells suspended in media (1 × 10^5^ in 1 ml) were pre-treated with 10 μM MG132 for 2 h, followed by co-treatment with DMSO or CPT-11 (15 or 30 μM) for 1 h. The mix was subsequently centrifuged at 1500 rpm and re-suspended in PBS containing 10 μM MG132, 400 μg/ml proteinase K (both from Sigma-Aldrich, Gillingham, UK) and DMSO or CPT-11 (15 or 30 μM). Cell suspension was subsequently mixed with 1.2% Type VII low-melt agarose warmed to 42°C (1:1 ratio) and the mix immediately layered onto frosted glass slides pre-coated with 0.6% agarose. Once set, the slides were incubated in lysis solution (pH 10; 400 μg/ml proteinase K, 10 μM MG132, CPT-11 (15 or 30 μM), 10 mM Tris–HCl, 100 mM EDTA pH 8.0, 1% Triton X-100, 1% DMSO) at 37°C for 3 h. Slides were then submerged in electrophoresis buffer (50 mM NaOH, 1 mM EDTA, 1% DMSO) for 45 min to allow for DNA unwinding prior to DNA separation by electrophoresis at 12 V for 25 min. On completion, slides were immersed in neutralisation buffer (0.4 M Tris–HCl; pH 7) for at least 1 h prior to staining with Sybr-Green diluted 1:10 000 in PBS for 10 min. Tail moment measurements for 100 cells per sample were obtained using the Comet Assay IV software (Perceptive Instruments, UK).

### Measurement of TOP1cc: CsCl fractionation and immunoblotting

TOP1 protein–DNA complexes (TOP1cc) were purified using caesium chloride density gradients. Cells (2 × 10^6^) were lysed in 1% sarcosyl, 8 M guanidine HCl, 30 mM Tris pH 7.5 and 10 mM EDTA. Cell lysates were incubated at 70°C for 15 min to remove all non-covalently bound proteins from DNA. Cell lysates were then loaded on a caesium chloride density (CsCl) step gradient (5 ml total volume) and centrifuged at 75 600 × g at 25°C for 24 h to separate free proteins from DNA. Ten consecutive 0.5 ml fractions were collected and slot blotted onto Hybond-C membrane (Amersham). To ensure equal DNA loading, the DNA concentration in each extract was determined fluorimetrically using PicoGreen (Molecular Probes/Invitrogen). Covalent TOP1–DNA complexes were then detected by immunoblotting with anti-TOP1 antibodies (sc-32736, Santa Cruz) and visualised by chemiluminescence. Fractions enriched for TOP1cc were pooled and subjected to serial dilution followed by band quantifications using the BioRad ChemiDoc platform.

### Cell cycle analysis

Fluorescence-activated cell sorting (FACS) analysis was used to determine cell cycle profiles. Sub-confluent monolayer cells were treated continuously with CPT-11 and collected at 24, 48 and 72 h post-treatment by trypsinisation, washed twice in ice-cold PBS and 4 × 10^6^ cells re-suspended in 0.5 ml PBS. The suspension was gently vortexed for 10 s to ensure separation of aggregated cells and added slowly to 4.5 ml ice-cold 70% ethanol for cell fixation, carried out at 4°C for at least 1 h and up to 48 h for the earliest timepoint. Cells were washed once in PBS and re-suspended in 1 ml PI staining solution to contain 0.1% Triton X-100, 10 μg/ml propidium iodide (Molecular Probes, Paisley, UK), 100 μg/ml RNase A (Sigma-Aldrich, Gillingham, UK). Cells were stained in the dark for 30 min at room temperature prior to cell sorting on the BD FACSCANTO system (BD Biosciences, Oxford, UK) and analysis using FCS Express 4 Flow software version 4.07.0011 (De Novo Software, Glendale, USA).

### DNA fibre assay

Exponentially growing cells were pulse-labelled with 25 μM CldU (Sigma) for 20 min followed by 250 μM IdU (Sigma) for 30 min to quantify replication fork speed. Cells were pulse-labeled with 25 μM CldU for 10 min followed by 30 min treatment with 50 μM CPT-11 (Sigma) then pulse-labelled with 250 μM IdU for 1 h to quantify replication fork structures. Cells were then washed and resuspended in cold PBS and diluted to ≈500 000 cell/ml. Each sample was spread on multiple Menzel-Glaser Superfrost slides, fixed, and stored at 4°C. Samples were then incubated in blocking solution (PBS + 1% BSA + 0.1% Tween-20) for 45 min followed by acid denaturation using 2.5 M HCl. Slides were stained with a mixture of Rat anti-BrdU (1:500; clone BU1/75; AbD Serotec) targeting CldU and Mouse anti-BrdU (1:500; clone B44; Becton Dickinson) targeting IdU. Cells were fixed using 4% paraformaldehyde and DNA fibres visualized by staining with Alexafluor 488 donkey anti-Rat and Alexafluor 555 goat anti-Mouse (Molecular Probes) secondary antibodies. Finally, slides were mounted in Fluoroshield (Sigma) and stored at −20°C. DNA fibres were examined using Olympus BX53 fluorescence upright microscope. Lengths of the fibres were measured, and speeds of the forks were determined using speed = length (Kb) / time (min). At least 238 fork structures were counted per sample where green-red signals represent on-going forks, green only represent stalled forks, and red only represent new origin firing.

### CellTiter-blue viability assay

Irinotecan sensitivity was determined using the CellTiter-Blue cell viability assay (Promega, Southampton, UK). Cells were seeded in triplicate at 3000 cells per well of a 96-well plate in irinotecan-containing media (0–20 μM). The plates were incubated at 37°C for 4 days prior to analysis with the CellTiter-Blue reagent. 20 μl reagent was added to each well, the plates incubated at 37°C for 1.5 h and fluorescence intensity data collected using the GloMax Multi Detection System (Promega, Southamption, UK) at excitation and emission wavelengths of λ_ex_ 560 nm and λ_em_ 590 nm. Growth fraction was calculated using background-subtracted readings as: (fluorescence read^treated^/fluorescence read^untreated^).

### Microarray expression profiling

The RNeasy mini RNA purification kit (Qiagen, Manchester, UK) was used to purify mRNA from the RKO parental cell line and the irinotecan-resistant clones RSC316 and RSC526 according to the manufacturers details. The mRNA quality was verified by measuring 260/280 and 260/230 ratios using a Nanodrop (Wilmington, USA). The mRNA samples were subsequently sent to Cambridge Genomic Services (CGS, Cambridge, UK) for additional quality control prior to microarray analysis using a HumanHT-12 v4 WG-GX Beadchip on the Illumina BeadArray system. The data was returned in the format X and the real-fold change in mRNA levels calculated using the equation 2^*X*^.

### Sister chromatid exchange

Exponentially growing cells were either mock-treated or treated with 1 μM CPT-11 for 1 h at 37°C. Cells were then washed twice with PBS and incubated in CPT-free media supplemented with 20 μM 5-bromodeoxyuridine (BrdU) for two cell generations (∼44 h). The mitotic inhibitor, colcemid (0.08μg/ml) was then added to the media and 1 h later, cells were pelleted and incubated in hypotonic solution of 37.5 mM KCl for 5 min at 37°C. Cells were fixed by dropwise addition of Carnoy's solution (methanol: acetic acid; 3:1 v/v) and incubated at −20°C for 16–24 h. Cells were then spread on cold wet slides that were pre-soaked in double distilled water containing Decon 90 for 2 days, and metaphase spreads were left to dry and aged at room temperature for 3 days. Slides were incubated in Hoechst 33258 for 12 min at room temperature in the dark, washed twice with distilled water, immersed in SSC buffer (2 M NaCl, 0.3 M tri-sodium citrate, pH 7), and exposed to 365 nm UV light for 15 min. Slides were subsequently incubated in SSC buffer for 60 min at 60°C and stained with 10% Giemsa solution in 0.05 M phosphate buffer pH 6.8. At least 40 metaphase spreads were scored for SCEs by Olympus BX51 microscope.

### Immunohistochemistry

A commercially available colon cancer tissue microarray (TMA) (Abcam, Cambridge, UK; catalogue number ab178133) was used to perform immunohistochemistry (IHC) as previously described (42). This TMA comprises 96 samples from 48 cases including primary (*n* = 48) and metastatic colorectal carcinoma (*n* = 36) in addition to normal human colon tissue (*n* = 12), which served as a positive control along with sections from a transitional cell carcinoma (TCC); the latter was obtained from the histopathology department archive. Tissue sections were deparaffinised in xylene and dehydrated in 100% ethanol followed by incubation in 3% methanolic H_2_O_2_ for 20 min to block endogenous peroxidase. Antigen retrieval was carried out by incubating the slides in buffer comprising 1 mM EDTA, 0.05% Tween20 and 1000 ml distilled water (pH 9.0) for 20 min at 95°C. Slides were washed in PBS, blocked with serum for 30 min and incubated with a rabbit monoclonal antibody for Histone H4K16ac (Abcam, catalogue number ab109463) at 4°C overnight in a humidified container (1:100 dilution). Omission of the primary antibody served as negative control. After overnight incubation, unbound primary antibody was washed off. A Vectastain Elite kit was used for secondary antibody and Avidin–Biotin Complex (ABC) at RT in accordance with the manufacturer's instructions (Vector laboratories, Burlingame, USA). Secondary antibody was added for 30 min followed by a wash and incubation with ABC for another 30 min. 3,3΄-diaminobenzidine (DAB) (Vector laboratories) was used to stain slides for 2 min and colouring reaction stopped using distilled water. Slides were counterstained with haematoxylin, dehydrated in graded alcohols and mounted using DPX mounting media and glass cover slips. Staining was considered positive when a brown nuclear reaction was observed. IHC staining was quantified using ImageJ software (version 1.49, NIH, USA) (43) and the IHC profiler plugin which is a recently developed and validated open source tool (44). Staining intensity and percentage positivity in each sample was obtained and a paired Student's *t*-test used to determine the statistical significance between normal, primary and metastatic carcinoma in addition to comparison between different tumour grades. A *P-*value of <0.05 was considered significant.

## RESULTS

**Figure 1.**
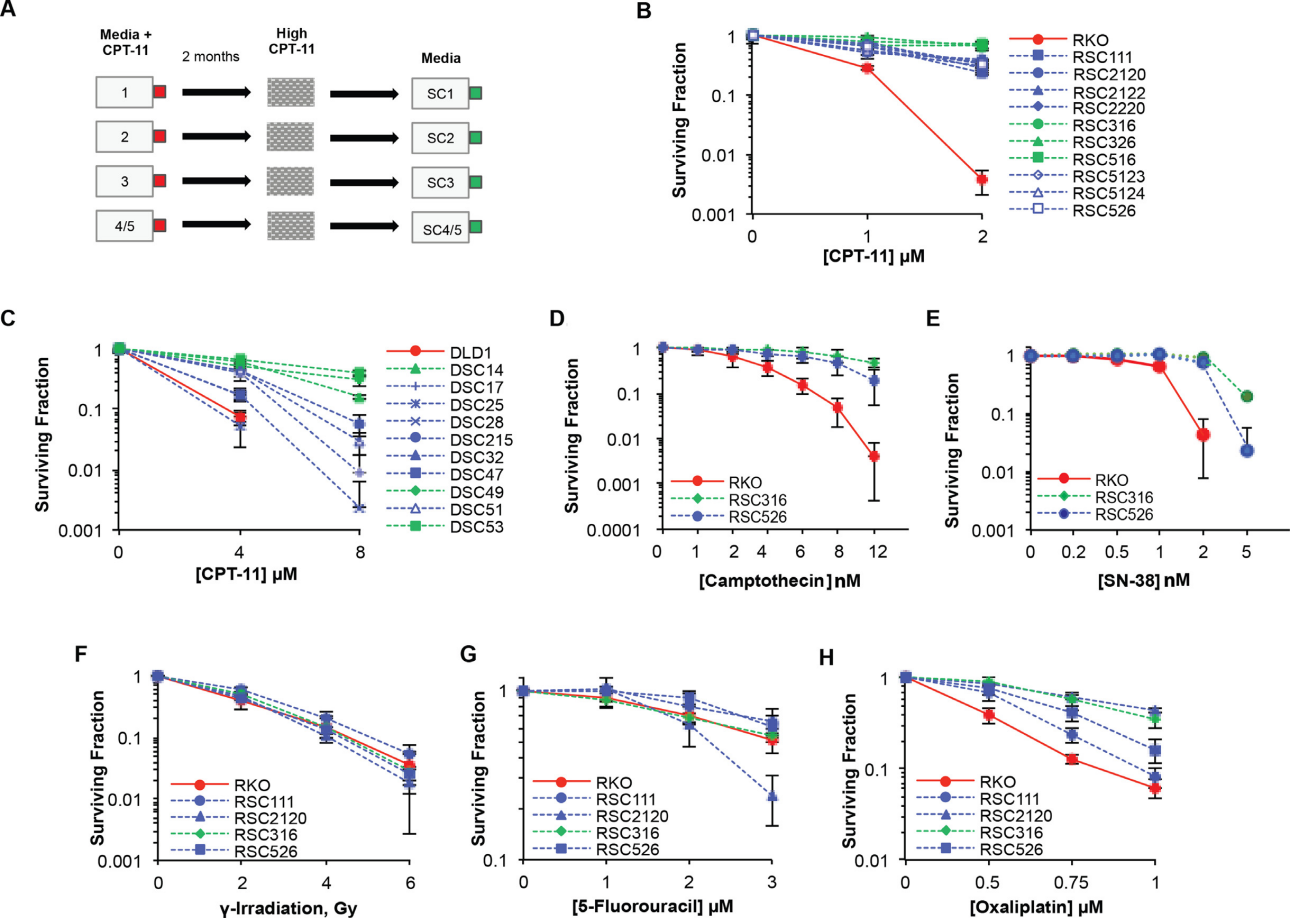
Irinotecan-resistant colorectal cancer cells are resistant to oxaliplatin but not to 5-fluorouracil or ionising radiation. (**A**) RKO and DLD1 cells were continuously treated with irinotecan (CPT-11) at a daily low dose (culture flask 1), daily moderate dose (culture flask 2), daily dose that increased at every split (culture flask 3) or a high dose that was repeated post-recovery (culture flask 4/5). After approximately 2 months, single cell dilution and CPT-11 selection was carried out to identify single CPT-11 resistant clones (first number represents flask of origin) that were stored and cultured in media lacking CPT-11 for further experimentation. Selected CPT-11 resistant clones derived from the parental RKO (**B**) and DLD1 (**C**) cell lines were seeded at low density and treated continuously with either 1 or 2 μM CPT-11 for the duration of colony formation (7–12 days). Colonies were fixed, stained, counted and the surviving fraction calculated as the surviving colony fraction^treated^/surviving colony fraction^untreated^ where surviving colony fraction = colonies counted/total cells seeded. Results are the average of 3 independent biological replicates ± STD. A survival assay was similarly carried out following camptothecin treatment (**D**), SN-38 (**E**), exposure to ionising radiation (**F**), 5-fluorouracil treatment (**G**) and oxaliplatin treatment (**H**). Data are the average of three independent biological replicates ± STD.

Drug resistance is an unmet clinical challenge hindering the success of camptothecin-based treatments such as irinotecan (CPT-11). To examine the mechanism of irinotecan resistance in colorectal cancer (CRC), we generated resistance models using two colorectal cancer cell lines, RKO and DLD1. RKO was previously shown to be sensitive to irinotecan whereas DLD1 was identified as relatively resistant (37). Cells were cultured in media containing varying concentrations of irinotecan (single dose and dose escalation) over a period of two months. Irinotecan-resistant clones were subsequently challenged with high irinotecan dose on single cell dilutions, followed by picking and expansion of surviving single colonies (Figure 1A). Once established, clones were grown and maintained in media lacking irinotecan, thereby enabling the study of resistance using the parental cell lines as controls. Using a clonogenic survival assay, all selected clones were confirmed to be irinotecan-resistant despite similar growth rates (Supplementary Figure S1A) and either fell into two distinct sub-populations or displayed a variation in the degree of acquired resistance (Figure 1B and C).

Irinotecan is often combined with other treatments in the clinic for colorectal cancer treatment such as radiation therapy or 5-fluorouracil (5-FU). Irinotecan-based treatment regimens are frequently replaced with oxaliplatin-based regimens when resistance emerges. We therefore first examined whether resistance to irinotecan also imparts resistance to others. Irinotecan-resistant clones were subjected to survival assays following exposure to γ-irradiation, oxaliplatin, 5-FU, SN-38 or camptothecin (CPT). A similar degree of resistance was observed following SN-38 or CPT treatment, consistent with their function as TOP1 poisons, ruling out differences in irinotecan conversion to its active form, SN38, as the cause of resistance (Figure 1D and E). In contrast, resistance was not observed following γ-irradiation or 5-FU (Figure 1F and G). However, all four clones displayed a similar degree of resistance to oxaliplatin as they did to irinotecan (Figure 1H). These experiments suggest that cross-resistance occurs between irinotecan and oxaliplatin but not between irinotecan and γ-irradiation or 5-FU.

**Figure 2.**
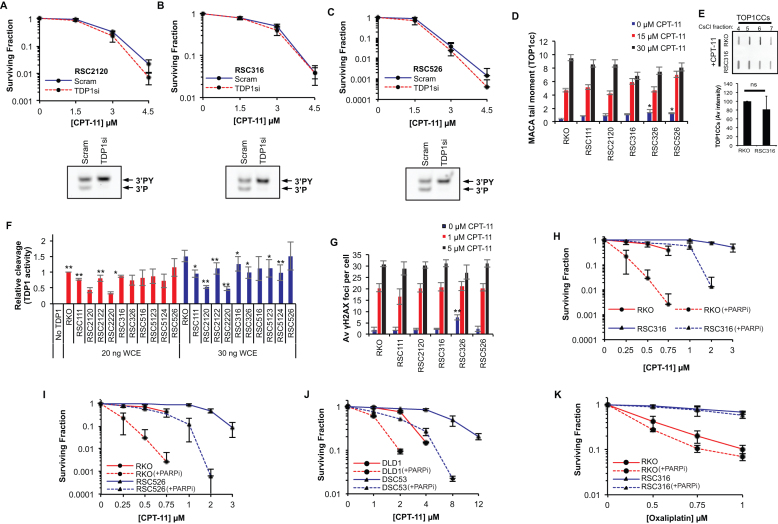
PARP1/TDP1 axis does not underpin irinotecan resistance in CRC cells. Irinotecan**-**resistant RSC2120 (**A**, upper panel), RSC316 (**B**, upper panel) and RSC526 (**C**, upper panel) were treated with a scrambled siRNA or a pool of four TDP1 siRNA sequences followed by treatment with the indicated concentrations of irinotecan. Survival was calculated from three biological replicates ± STD. Lysates prepared from the corresponding TDP1-depleted cells were subjected to a TDP1 activity assay and reaction products separated by 20% denaturing PAGE (A–C, lower panels). The substrate (3΄PY) and product (3΄P) are indicated for one representative experiment of three. (**D**) Parental RKO and RKO derived irinotecan-resistant cells were treated with irinotecan for 1 h and TOP1 cleavage complexes (TOP1cc) quantified by the modified alkaline comet assay. The average tail moment of three biological replicates is shown ± STD for 100 cells measured per sample. (**E**) Cells were treated with irinotecan and TOP1cc purified by CsCl fractionation followed by immunoblotting using anti TOP1 antibodies. CsCl fractions enriched with TOP1cc (fractions 4–7) are shown from a representative experiment (*top*) and the average intensity of TOP1cc ± STD from three biological replicates is shown (*bottom*). (**F**) Whole cell extract prepared from RKO derived irinotecan resistant clones were incubated with oligonucleotides harbouring a 3΄-phosphotyrosine modification (3΄PY) and 5΄-fluorophore to monitor TDP1 catalytic activity. Reaction products were separated by 20% denaturing PAGE and imaged using a FujiFilm Fluor Imager FLA-5100 at 635 nm. TDP1 catalytic activity was quantified as % cleavage of 3΄PY to 3΄P and data are the average of three biological replicates ± STD. (**G**) The indicated cells were treated with DMSO, 1 μM or 5 μM CPT-11 for 1.5 h and left to recover for 2 h prior to fixing, permeabilising and immunostaining with a γH2AX primary antibody (JBW301, Millipore) and a FITC-labeled secondary antibody. γH2AX foci were counted for 36 cells per sample on a Nikon Eclipse e-400 microscope and the average number of γH2AX foci per cell for three independent experiments is shown ± STD. The RKO cell line and resistant clones RSC316 (**H**) and RSC526 (**I**) as well as the DLD1 cell line and resistant clone DSC53 (**J**) were subjected to an irinotecan survival assay in the presence and absence of 1 μM olaparib (PARP inhibitor). The cells were pre-treated with olaparib for 1 h prior to addition of indicated amounts of CPT-11, both left in for the duration of colony formation. The colonies were fixed, stained and counted and presented as the average surviving fraction for three independent experiments ± STD. (**K**) Similarly, the RKO and RSC316 cell lines were subjected to an oxaliplatin survival assay in the presence and absence of 1 μM olaparib. The surviving fraction is shown for the average of three independent experiments ± STD.

TDP1 and TOP1 levels are key determinants of irinotecan response in colorectal cancer cells and TDP1 depletion has been shown to sensitise the RKO cell line to irinotecan by increasing the number of cytotoxic DSBs (37,45). We therefore examined if TDP1 depletion could re-sensitise CRC resistant clones to irinotecan. TDP1 was depleted from three separate resistant clones using a pool of four siRNA sequences followed by irinotecan clonogenic survival assays. In a marked contrast to the parental RKO cells, for which we previously reported irinotecan sensitisation upon TDP1 depletion (37 and Supplementary Figure S1B), irinotecan-resistant cells were also resistant to TDP1 depletion (Figure 2A–C, upper panels). These observations were further confirmed in two additional resistant clones (Supplementary Figure S1C). The lack of sensitisation was not due to inefficient TDP1 depletion since the product of TDP1 catalytic activity was readily detectable in an *in vitro* assay from lysates of cells treated with scrambled siRNA but not with TDP1 siRNA (Figure 2A–C, lower panels and Supplementary Figure S1C and S1D). We conclude from these experiments that TDP1 manipulation is unlikely to be effective in overcoming irinotecan resistance in CRC.

Next, we examined whether TDP1 and TOP1 levels or activity were altered. Western blotting of whole cell extracts revealed no detectable gain of TDP1 or loss of TOP1 expression between parental and resistant clones (Supplementary Figure S2A and S2B). We assessed TOP1 function by measuring the products of TOP1 activity (TOP1cc) that accumulate following CPT-11 treatment using the modified alkaline COMET assay (MACA) (22). No significant difference was observed in the levels of TOP1cc upon irinotecan treatment for all five irinotecan-resistant RKO clones tested compared to the parental RKO cell line (*P* > 0.05; *t*-test), indicating no detectable change in TOP1 activity (Figure 2D). This was further confirmed by an independent assay in which we purified irinotecan-induced TOP1cc by CsCl fractionation followed by anti-TOP1 immunoblotting (Figure 2E). Upregulation of TDP1 was ruled out as a mechanism of resistance as measured by TDP1 activity assay, which is a more sensitive readout than immunoblotting (Figure 2F and Supplementary Figure S2C). Since TDP2, a TOP2 PDB repair factor (46), has also been shown to contribute to TOP1 PDB repair under certain circumstances (47,15), we compared parental and resistant cells for sensitivity to TOP2 targeting drugs and TDP2 expression. Irinotecan-resistant cells did not show cross-resistance to doxorubicin or etoposide and exhibited comparable expression of TDP2 (Supplementary Figure S3A and S3B). Furthermore, *in vitro* TDP1 substrate processing, which should also reveal TDP2 activity on TOP1 substrates, was lost in the absence of TDP1, suggesting that TDP2 is not compensating for repair in irinotecan-resistant cells (Figure 2A–2C, lower panels and Supplementary Figure S1C and S1D). The key cytotoxic lesion generated by trapped TOP1cc and subsequent formation of a PDB is a DNA double-strand break (DSB) (20,48). RKO cells and five irinotecan-resistant clones displayed no significant difference in the average number of γH2AX foci per cell (p>0.05, t-test), a cellular marker of DNA DSBs (Figure 2G). The lack of difference was not due to drug saturation since a lower non-saturating dose (1 μM) of irinotecan also failed to reveal a difference in DSB levels. This finding additionally implies that drug processing and drug efflux are unlikely to account for resistance, which is supported by the variable expression of the drug efflux factor MRP4 across multiple resistant clones (Supplementary Figure S3C). Together, we conclude from these experiments that a reduction of TOP1 expression/activity, an increase in TDP1 or TDP2 expression/activity or rate of DSB formation does not account for irinotecan resistance.

We next examined whether resistance could be overcome by the FDA approved PARP inhibitor, olaparib. Treatment with 1 μM olaparib was able to restore irinotecan sensitivity of resistant cells (Figure 2H and 2I, and Supplementary Figure S4A and S4B) without affecting plating efficiencies or survival in irinotecan untreated cells (Supplementary Figure S4E, S4F and S4G). Whilst the extent of sensitisation in the resistant cells was comparable to the inherent irinotecan sensitivity of the parental RKO cell line, olaparib similarly sensitised the parental RKO cells to irinotecan. This sensitisation was also observed in the DLD1 parental cell line and the DLD1-derived resistant clones, DSC53 and DSC215 (Figure 2J, Supplementary Figure S4C). PARP inhibition did not sensitise the clones to oxaliplatin, as would be expected if a PARP related resistance mechanism was shared (Figure 2K and Supplementary Figure S4D). We conclude from these experiments that olaparib is able to promote irinotecan sensitivity in both parental and resistant CRC cells and thus cannot provide a mechanism for resistance. The ‘immunity’ of irinotecan resistant cells to TDP1 depletion further confirms that changes in the TDP1/PARP1 axis cannot explain resistance.

**Figure 3.**
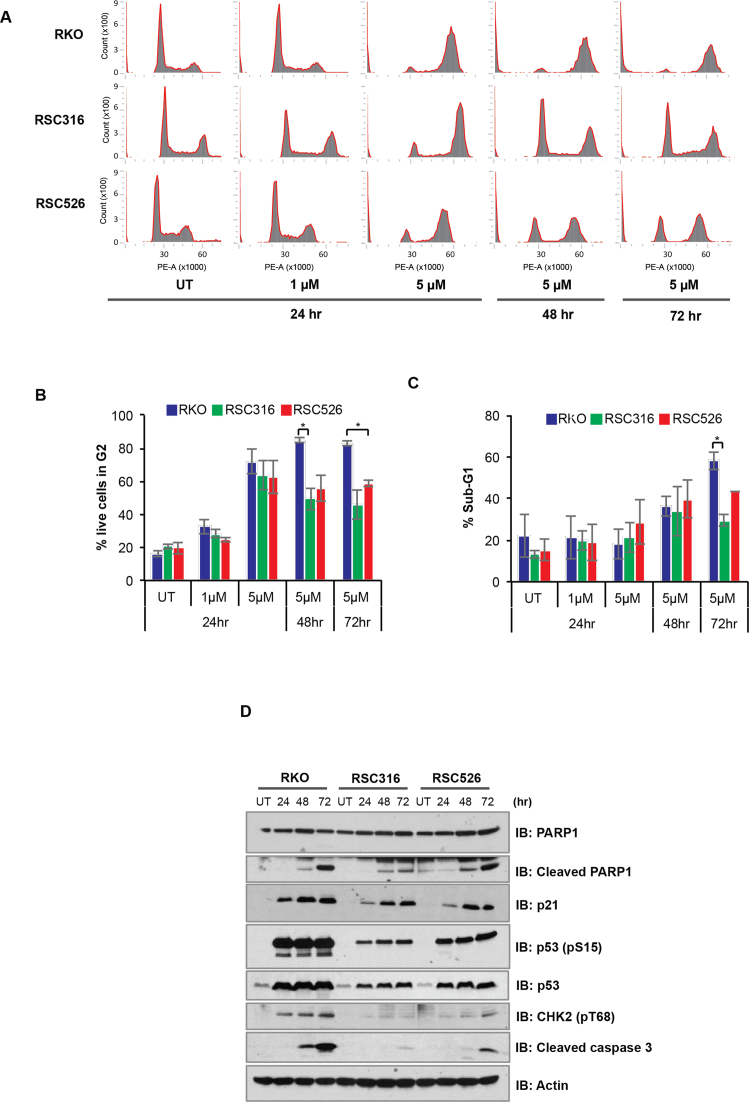
Irinotecan-resistant cells display attenuated cell cycle arrest and apoptosis following irinotecan treatment. (**A**) The RKO parental, RSC316 and RSC526 resistant cells were treated continuously with 1 μM or 5 μM CPT-11 for 24, 48 and 72 h. Cells were stained with propidium iodide solution and DNA content analysed by cell sorting on a FACS CANTO system. Cell cycle distribution was determined using the FCS Express 4 Flow software and representative cell cycle charts for one of three biological replicates is shown. (**B**) The proportion of live cells in G2 was determined by FACS analysis and data represent the average of three biological repeats ± SEM. (**C**) The average percentage of sub-G1 cells obtained by FACS analysis are graphically depicted for three biological repeats ± SEM. (**D**) Whole cell extract prepared from the indicated cell lines treated continuously with 5 μM CPT-11 for 24, 48 and 72 h were separated by 10% SDS-PAGE and analysed by immunoblotting using anti PARP1 and cleaved PARP1 (A6.4.12, Biorad), p21 (SC417, Santa Cruz Biotechnologies), p53 phosphorylated at serine 15 (#9284, Cell Signaling Technology), cleaved caspase 3 (E83-77, Abcam), pT68 CHK2 (#2661, Cell Signaling Technology), p53 (DO-1, Sigma-Aldrich) and actin (A4700; Sigma-Aldrich). A representative blot for one of three repeats is shown.

DNA DSBs generated by CPT-based agents signal cellular G2/M arrest whilst overwhelming levels of unrepaired DNA DSBs activate apoptosis-mediated cell death (35). To examine G2/M checkpoint we compared parental and resistant clones for cell cycle profile by fluorescence-activated cell sorting (FACS). The parental cell line and resistant clones experienced G2/M arrest at the 24 h time point (Figure 3A and B). However, whilst the parental RKO cell line remained fully arrested during subsequent 48 and 72 h incubations with irinotecan, resistant cells were able to partially overcome G2/M arrest (Figure 3B, *P* < 0.05 *t*-test). Notably, the more resistant RSC316 cells were more able to overcome G2/M arrest compared to the less resistant RSC526 cell line. Furthermore, resistant clones exhibited fewer ‘dead’ cells (sub-G1) compared to the parental cell line (Figure 3C). These observations show that the resistant clones are less able to maintain G2/M arrest compared to parental cells, which may be concomitant with a reduced ability to trigger apoptotic response following irinotecan treatment. Consistent with this, parental and resistant clones possessed an irinotecan-dependent increase in levels of cleaved PARP1, p21, p53 phosphorylation and cleaved caspase 3 in line with an induction of p53, however the induction was much more pronounced in the parental RKO cell line than resistant clones (Figure 3D). In agreement with the cell cycle analysis, the apoptotic response appears to be more attenuated in the more resistant RSC316 clone compared to the less resistant RSC526 clone. Altogether, these findings demonstrate that the resistant clones possess intact cell cycle arrest and apoptotic responses following irinotecan treatment, however they are less able to maintain the arrest than the parental cell line.

**Figure 4.**
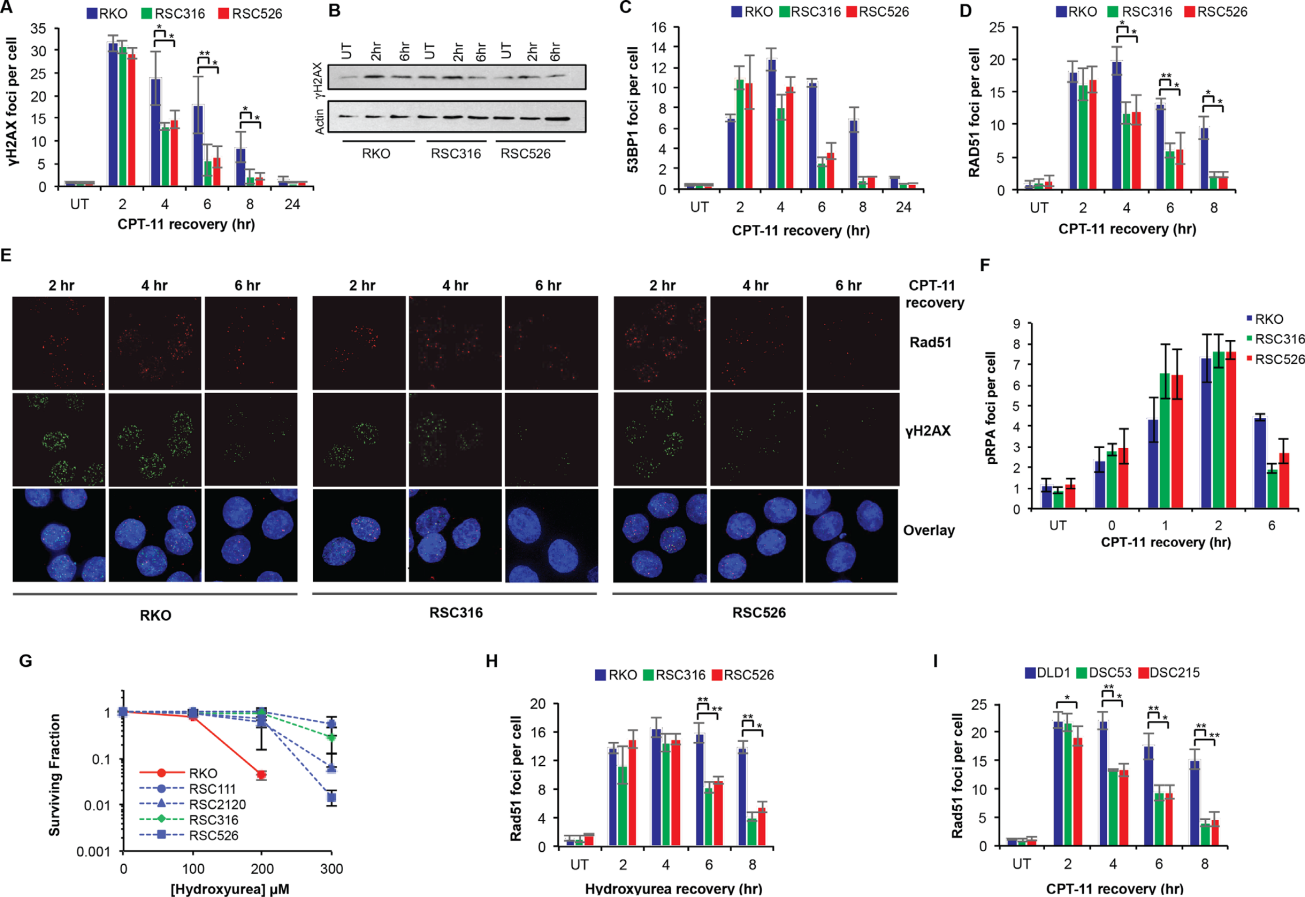
Improved double-strand break repair in irinotecan-resistant cells. Parental RKO and RKO-derived irinotecan-resistant RSC316 and RSC526 cells were exposed to DMSO ‘UT’ or 2 μM CPT-11 for 1.5 h and left to recover in CPT-11 free media for 2, 4, 6, 8 and 24 h. Cells were subsequently fixed and permeabilised for immunostaining with antibodies against γH2AX (JBW301, Millipore) (**A**) or cell lysates were fractionated by SDS PAGE and analysed by immunoblotting (**B**). Cells treated as in (A) were additionally immunostained with antibodies against 53BP1 (A300-272A, Bethyl Laboratories) (**C**), RAD51 (H-92, Santa Cruz Biotechnologies) (**D**), or pRPA (A300-245A, Bethyl Laboratories, Texas, USA) (**F**). The number of foci per cell for 36 cells was counted manually on a Nikon Eclipse e-400 microscope and the average number of foci per cell for three independent experiments is shown ± STD. Asterisks denote statistical significance; *P < 0.05, **P < 0.01; Student's *t*-test. (**E**) Representative images for RAD51 and γH2AX staining are shown for the indicated cell lines and recovery timepoints. (**G**) RKO parental and its irinotecan-resistant derived clones were incubated with the indicated concentrations of hydroxyrea (HU). Survival was calculated as described in Figure 1 and data are the average of three independent biological repeats ± STD. (**H**) The parental RKO cell line and the RSC316 and RSC526 irinotecan resistant clones were treated in media containing DMSO ‘UT’ or 0.5 mM HU for 1.5 h and left to recover for the indicated timepoints. Cells were fixed, permeabilised and immunostained using antibodies against RAD51 as above. The number of foci per cell for 36 cells was counted manually and the average number of foci per cell for three independent repeats is shown ± STD. (**I**) The DLD1 parental cell line and DLD1 derived irinotecan-resistant DSC53 and DSC215 cells were treated with 4 μM CPT-11 for 1.5 h, washed and left to recover in CPT-11 free media for indicated timepoints. Cells were subsequently fixed, permeabilised and immunostained with antibodies against RAD51 as above. Average RAD51 foci per cell for 36 cells counted per experiment is shown for three independent biological repeats ± STD. Asterisks denote statistical significance; *P < 0.05, **P < 0.01; Student's *t*-test.

We next considered that the ability to overcome DSB-mediated cell cycle arrest and to attenuate apoptotic response could be due to increased DSB repair rates. To test this, cells were treated with irinotecan and DSB repair kinetics was monitored in subsequent incubations in irinotecan-free media for 2, 4, 6, 8 and 24 h. Consistent with results in Figure 2G, no difference in the number of γH2AX foci was observed at the 2-h time point, however, at later time points the number of γH2AX foci declined more quickly in resistant clones compared to parentals (Figure 4A, B and E). The increased ability of resistant clones to repair DSBs was further confirmed by 53BP1 immunostaining as an alternative marker for DSBs (Figure 4C). DSBs are repaired by homologous recombination (HR) or non-homologous end joining (NHEJ). The majority of breaks generated by IR are repaired by NHEJ and since we did not observe cross-resistance to IR (Figure 1F), we reasoned that improved NHEJ is unlikely to account for the increased repair rates. Consistent with this, depletion of the key NHEJ factor KU70 did not reverse irinotecan resistance (Supplementary Figure S5). Consequently, we examined if more efficient HR underlies resistance. To test this, we monitored RAD51 foci formation and clearance as a specific marker of HR (49,50). Although RAD51 foci formed with similar kinetics and frequency during the first 2 h following irinotecan treatment in all clones tested, the rate at which they declined was significantly faster in resistant clones (Figure 4D and E; *P* < 0.05, *t*-test). The rate of pRPA foci clearance was also consistently faster in resistant clones compared to parentals (Figure 4F). If upregulation of HR accounts for resistance to irinotecan, one would expect cross-resistance to specific HR targeting therapies. To test this, we treated cells with the replication inhibitor hydroxyurea (HU) and examined cellular response using clonogenic survival assays. Indeed, irinotecan-resistant clones were also resistant to HU (Figure 4G). Furthermore, Rad51 foci clearance was significantly faster following HU treatment in the resistant clones compared to parental (Figure 4H; *P* < 0.05, *t*-test). We subsequently examined two resistant clones derived from the DLD1 parental cell line; one highly resistant DSC53 and a slightly less resistant DSC215 clone. In striking similarity to observations with RKO-derived resistant cells, DLD1-derived resistant clones were able to clear RAD51 foci at significantly faster kinetics compared to the parental DLD1 cell line (Figure 4I). Notably, the observed cross-resistance with oxaliplatin (Figure 1H) could also be explained by an increase in HR since oxaliplatin-mediated damage is repaired in part by HR (51). Together, these data demonstrate that improved HR-mediated PDB repair underpins irinotecan resistance in CRC cells.

**Figure 5.**
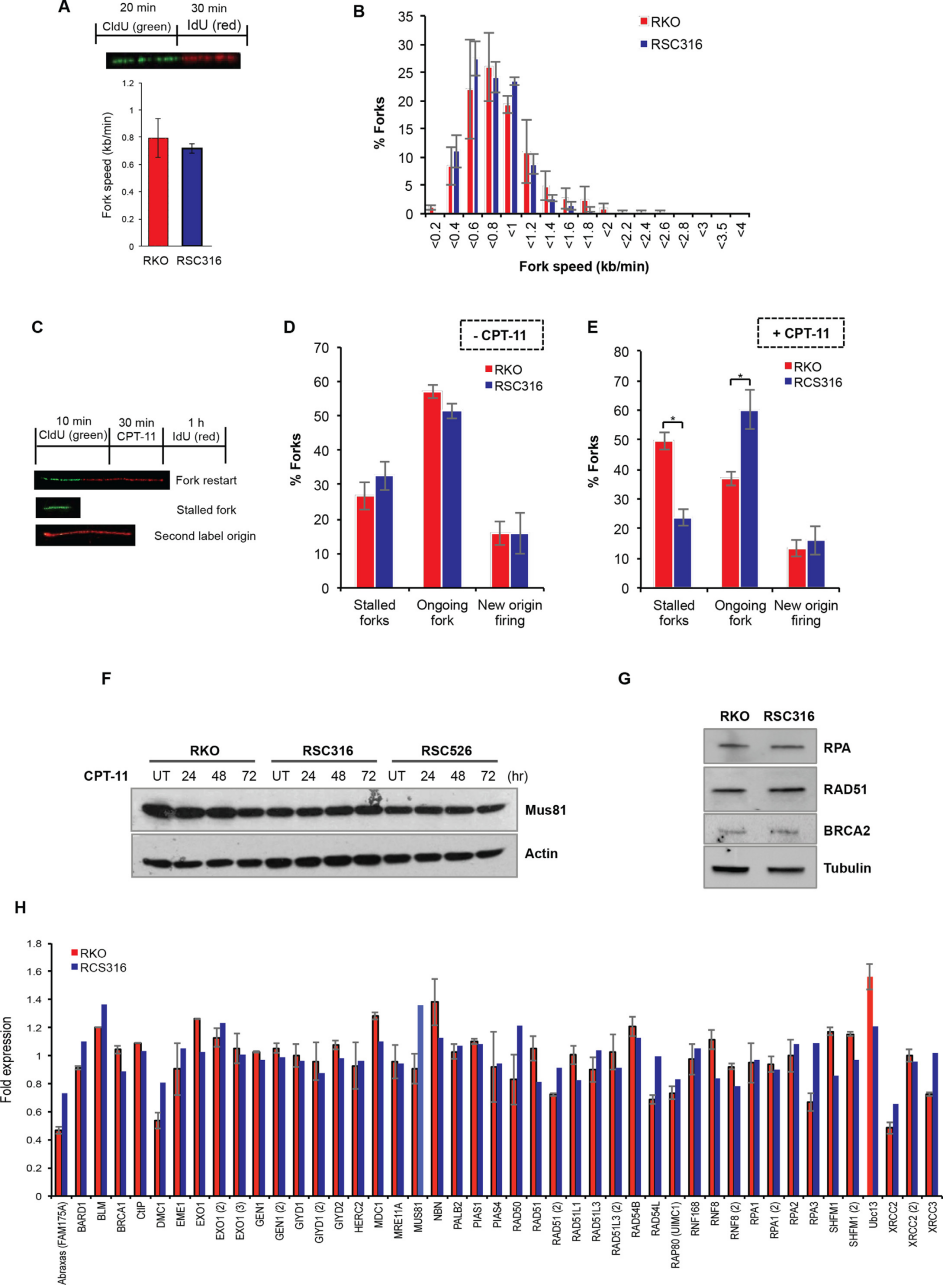
Irinotecan-resistant cells display more fork restart events but no detectable change in HR factors. (**A**) Parental RKO and its derivative RSC316 irinotecan-resistant cells were pulse labeled with CldU for 20 min to label newly replicated DNA, followed by IdU for 30 min to measure replication fork speed. DNA fibres were spread on superfrost slides, fixed, and stored at 4°C. DNA fibers were subjected to acid denaturation using 2.5 M HCl. Slides were then stained with a mixture of rat anti-BrdU (clone BU1/75, Serotec) targeting CldU and mouse anti-BrdU (clone B44; Becton Dickinson) targeting IdU. DNA fibres were visualised using donkey anti-rat Alexafluor 488 and goat anti-mouse Alexafluor 555 secondary antibodies on an Olympus BX53 fluorescence upright microscope. Lengths of the fibres were measured, and speeds of the forks were determined using speed = length (kb)/time (min). Data are the average of three biological replicates ± SEM. *Inset*: Schematic depicting the DNA labelling procedure. (**B**) The average distribution of fork speeds is depicted from three biological replicates ± SEM. (**C**) A schematic showing the labelling procedure and the different replication fork structures; green-red signals represent on-going forks, green only represent stalled forks, and red only represent new origin firing. (**D** and **E**) Parental RKO and its derivative irinotecan-resistant cells were pulse labelled with CldU for 10 min to label newly replicated DNA, followed by treatment with DMSO ‘-CPT-11’ (**D**) or 50 μM CPT-11 ‘+CPT-11’ (**E**) for 30 min, followed by 1 h incubation with IdU. DNA fibres were spread on superfrost slides, fixed, and processed as described in ‘A’. At least 238 fork structures were counted per sample and the average number of stalled forks, fork restart ‘ongoing forks’, and new origin firing was quantified from three biological replicates ± SEM. Asterisks denote statistical significance; *P < 0.05, Student's *t*-test. (**F** and **G**) Cell lysates from the indicated cell lines were fractionated by SDS-PAGE and analysed by immunoblotting. (**H**) Parental and resistant cells were exposed to CPT and mRNA extracted using RNeasy mini RNA purification kit. mRNA was subjected to microarray analysis using a HumanHT-12 v4 WG-GX Beadchip on the Illumina BeadArray system. Expression changes of HR genes in resistant cells were presented as fold change relative to the parental RKO cells. Data are the average of two independent biological repeats ± range.

In addition to its canonical roles during HR, RAD51 has recently been shown to promote replication fork reversal (18). Replication fork reversal can protect forks following TOP1 poisoning in a PARP1 dependent manner (17). Neither PARP1 depletion by siRNA nor depletion of the RECQ1 helicase that controls fork restart/reversal restored sensitivity to irinotecan in resistant clones, suggesting that changes in replication fork reversal is unlikely to account for irinotecan resistance (Supplementary Figure S6). An increase in DSB repair rates should however improve the ability to restart irinotecan-arrested replication forks. To test this, we first compared resistant and parental clones for replication fork speed using the DNA fibre assay (52). Cells were incubated with chlorodeoxyuridine (CldU) for 20 min to label newly replicated DNA followed by a 30 min incubation with iododeoxyuridine (IdU) (Figure 5A, inset). Parental RKO and resistant RSC316 cells exhibited comparable average fork speed of 0.79 and 0.72 kb/min, respectively (Figure 5A). Consistent with this, both cell lines did not show detectable difference in the distribution of fork rates (Figure 5B). We next compared replication fork structures by incubating cells with CldU to label newly replicated DNA followed by a challenge with CPT-11 and then incubation with iododeoxyuridine (IdU) (Figure 5C). Although parental and resistant cells showed similar frequencies of stalled and on-going forks (Figure 5D), treatment with irinotecan resulted in a significant difference in fork structures. Whereas parental RKO cells possessed ∼50% stalled forks and ∼35% fork restarts, resistant RSC316 cells possessed ∼25% stalled forks and 60% fork restarts (Figure 5E). The improved rate of fork restart is likely related to faster repair rates of DSBs by HR as suggested by a hyper-recombination phenotype (Supplementary Figure S7). We next examined if the observed increase in repair rate is driven by increased activity of the HR machinery *per se*. Together with key HR repair factors, a number of additional nucleases have been implicated during HR-mediated repair of CPT induced DNA damage such as MUS81 and XPF/SLX4 (6). Using immunoblotting, microarray and quantitative PCR, we observed no upregulation in the level of these nucleases nor in ATM signalling or the key HR repair factors CtIP, Rad51, BRCA1 and RPA (Figure 5F–H and Supplementary Figure S8). Depletion of the HR resection enzyme CtIP sensitised cells to irinotecan but did not selectively restore sensitivity in irinotecan-resistant cells (Supplementary Figure S9). Furthermore, mRNA profiling by microarray of parental and resistant cells failed to detect any significant change in the transcripts of genes known, or predicted, to participate in TOP1 mediated PDB repair (Supplementary Figure S10).

**Figure 6.**
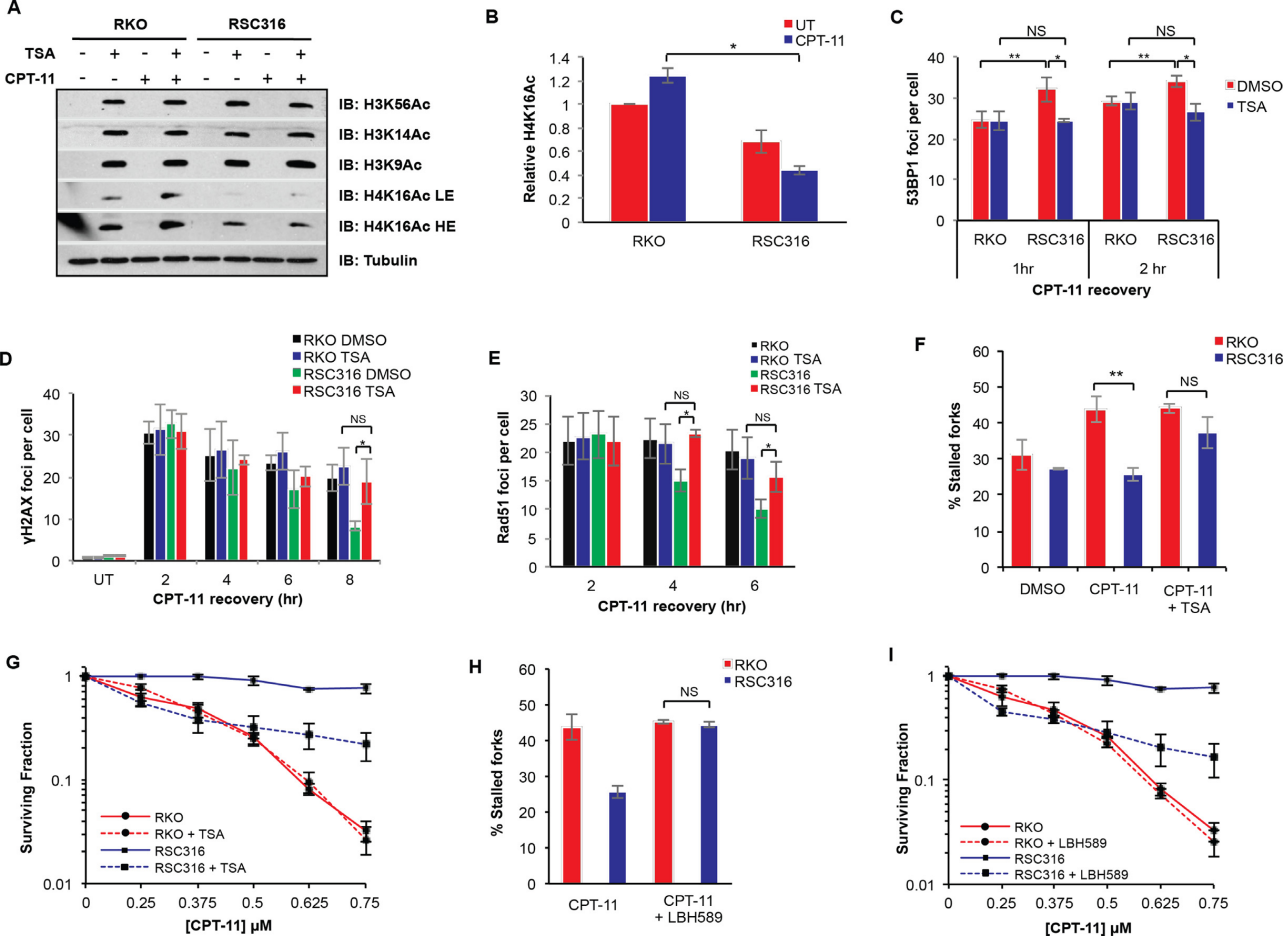
Deregulation of histone H4K16 acetylation in resistant cells promotes DNA repair and highlights the utility of HDAC inhibitors for the reversal of irinotecan resistance. (**A**) RKO and RSC316 cells were pre-treated with 1 μM TSA for 2 h, followed by treatment with 2 μM CPT-11 for 1.5 h and recovery for 2 h. Prepared whole cell extracts were separated by 15% SDS-PAGE and analyzed by immunoblotting using antibodies against H3K56Ac, H3K14Ac, H3K9Ac, H4K16Ac and tubulin. Representative blots for one of three repeats are shown. LE; low exposure, HE; high exposure. (**B**) Relative H4K16Ac expression levels were quantified from the TSA treated samples from 3 biological replicates, a representative of which is shown in (A) using ImageJ and subsequent normalizing to tubulin levels. Calculations from three replicates ± STD is shown. (**C**) RKO and RSC316 cells were pre-treated with 1 μM TSA for 2 h, followed by treatment with 2 μM CPT-11 for 1.5 h and recovery for 1 or 2 h. Cells were fixed, permeabilised and analysed by immunostaining for 53BP1. The number of foci per cell for 36 cells was counted manually on a Nikon Eclipse e-400 microscope and the average number of foci per cell for three independent experiments is shown ± STD. Cells were similarly treated with TSA and CPT-11 and allowed to recover for indicated timepoints for analysis of γH2AX (**D**) and Rad51 (**E**) foci per cell, measured as above. Results show the average of three biological replicates ± STD. RKO and RSC316 cells were pre-treated with 500 nM TSA (**F**) or 250 nM Panobinostat (LBH-589) (**H**) for 30 min, pulse labelled with CldU for 10 min to label newly replicated DNA, followed by treatment with 50 μM CPT-11 for 30 min, followed by 1 h incubation with IdU. DNA fibres were spread on superfrost slides, fixed, and processed. At least 50 fork structures were counted per sample and the average percentage stalled forks was quantified from three biological replicates ± SEM. Cells were treated with 10 nM of TSA (**G**) or 2.6 nM Panobinostat (LBH-589) (**I**), followed by exposure to the indicated concentrations of CPT-11 for the duration of colony formation. Survival was calculated from three biological replicates ± STD. Asterisks denote statistical significance; *P < 0.05, **P < 0.01; Student's *t*-test.

We next considered the possibility that changes in the chromatin landscape might underlie the improved PDB repair rate in resistant cells. Recent studies have highlighted the importance of chromatin modification in modulating the recruitment of DNA repair factors to damage sites. For instance, histone H4K16 acetylation appears to regulate the accumulation of the 53BP1 repair factor at DNA DSBs, thereby controlling repair dynamics (53). Whilst we found no detectable changes in PDB repair factors, we did however note faster but transient accumulation of the 53BP1 repair factor in response to irinotecan in the resistant clones (Figure 4C). This observation prompted us to compare parental and resistant cells for levels of H4K16 acetylation. RKO and RSC316 cells were pre-treated with the histone deacetylase (HDAC) inhibitor trichostatin A (TSA) followed by irinotecan treatment and recovery. Cell extracts were analyzed by western blotting using antibodies against H4K16Ac as well as other histone acetylation modifications including H3K14Ac, H3K56Ac and H3K9Ac (Figure 6A). Whilst no difference was observed in H3K14Ac, H3K56Ac and H3K9Ac between parental and resistant cells, the latter possessed remarkably lower levels of H4K16Ac, highlighting a reduction of H4K16Ac steady state levels (Figure 6B). This appears to be common across multiple resistant clones (Supplementary Figure S11A and S11B). These observations suggest that the turnover rate of H4K16Ac is perturbed in irinotecan-resistant cells, potentially explaining the increased 53BP1 accumulation to damage sites. If this is true, one would predict that increasing the level of H4K16Ac by pre-treatment with TSA could restore normal levels of 53BP1 accumulation. To test this, we carried out 53BP1 immunostaining on cells treated with irinotecan in the presence of TSA to enrich for the H4K16Ac modification. Indeed, the attempts to re-adjust the levels of H4K16Ac using TSA slowed 53BP1 accumulation to damage sites in both the RSC316 and RSC526 resistant clones, but not parental CRC cells, without affecting the initial number of γH2AX foci (Figure 6C, Figures S11C and S12B).

We next examined if restoring H4K16Ac would impact the DSB repair rate observed in irinotecan resistant cells. TSA treatment suppressed the faster rate of irinotecan-induced DSB repair in irinotecan resistant but not parental cells (Figure 6D). Following 8 h recovery, TSA had no significant impact on the number of γH2AX foci per cell in parental cells whereas an increase was observed in the RSC316 resistant cells from ∼7 to 20 foci per cell, which is similar to the number observed in parental cells. A similar result was obtained for the RSC526 clone (Supplementary Figure S12A). TSA had no detectable impact on the rate of RAD51 foci clearance in RKO parental cells but it significantly suppressed the faster clearance rate in the RSC316 resistant cells, bringing Rad51 foci per cell at 6 h recovery time point to a level similar to that observed in parental cells (Figure 6E). Consistently, TSA was also able to suppress the improved ability to re-start arrested forks in irinotecan resistant cells (Figure 6F). To further show that the phenotypes observed in our resistant clones is related to the slower turnover of H4K16Ac, we attempted to create irinotecan resistance in the parental RKO cell line by reducing H4K16Ac through depletion of one of the damage response H4K16 histone acetyl transferases (HATs), TIP60. Depletion of Tip60 moderately reduced the turnover of H4K16Ac in RKO cells (Supplementary Figure S11D). Clonogenic survival assays and growth assays show that Tip60 depletion partially promotes irinotecan resistance in the parental RKO cell line (Supplementary Figure S11E and S11F). Tip60 depletion consistently led to a mild increase in 53BP1 accumulation and faster γH2AX foci clearance (Supplementary Figure S11G and S11H).

**Figure 7.**
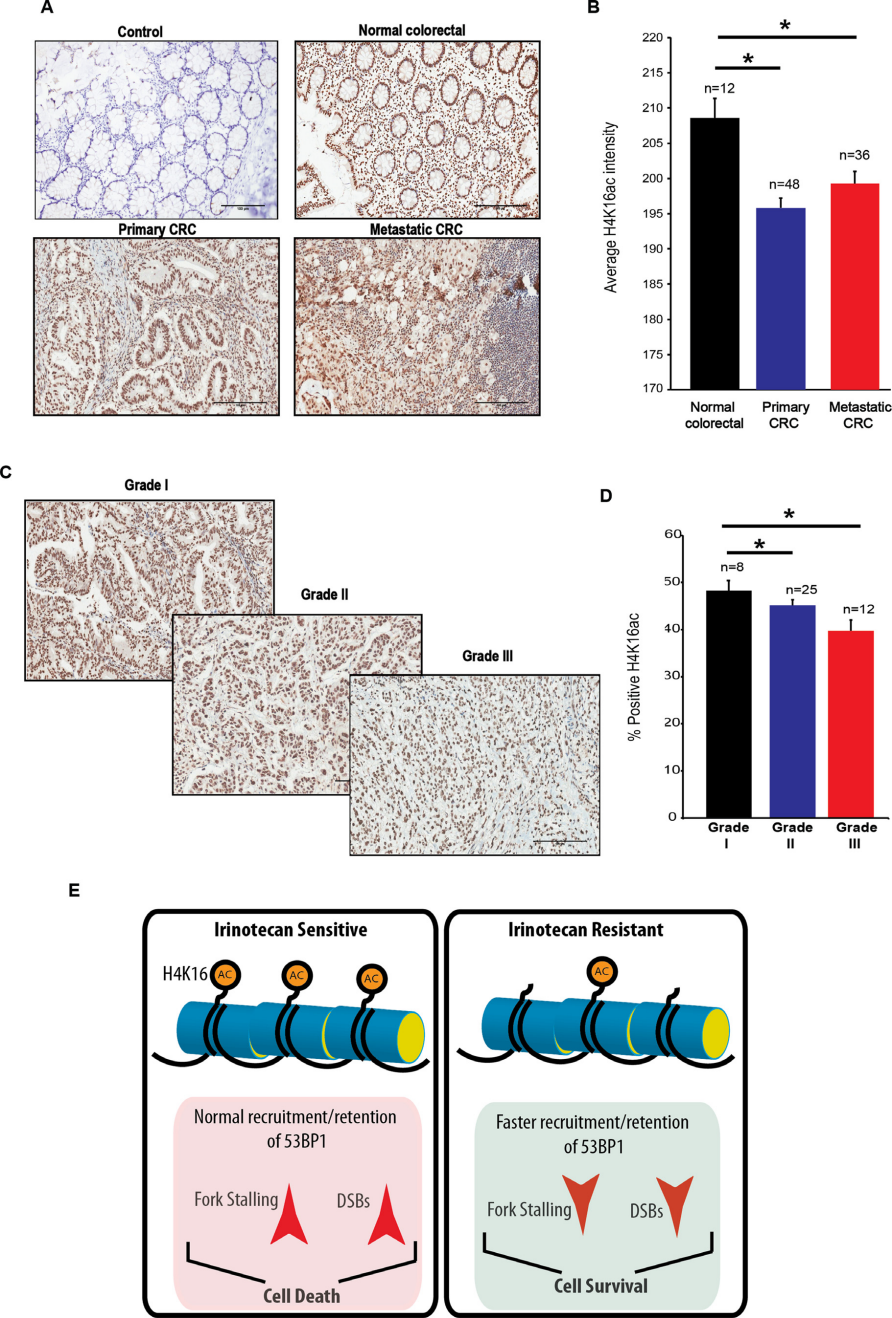
Hypoacetylation of H4K16 is a hallmark of CRC tissues. (**A**) Normal, primary and metastatic CRC tissues were subjected to immunohistochemical analyses using H4K16ac antibodies (Abcam, catalogue number ab109463) and a Vectastain Elite kit was used for secondary antibody and Avidin-Biotin Complex (ABC) conjugation. Staining was considered positive when a brown nuclear reaction was observed. A representative image from each condition is shown. Control; IHC was conducted without the incubation of primary antibodies. (**B**). IHC staining was quantified using ImageJ software (version 1.49, NIH, USA) and presented as average ± SEM. A paired Student's *t*-test was used to determine the statistical significance between normal, primary and metastatic carcinoma. Asterisks denote *P* < 0.05. CRC tissues with the indicated grades were subjected to IHC analyses as described above and a representative image is shown (**C**) and % positive H4K16ac was quantified and presented as average ± SEM (**D**). (**E**) A model for chromatin acetylation driven irinotecan resistance. In normal irinotecan-responsive cells, appropriate acetylation dynamics of H4K16 results in normal recruitment and retention of 53BP1, resulting in increased fork stalling and DSB persistence, ultimately causing cell death. Failure to maintain the appropriate dynamics of H4K16 acetylation results in increased accumulation of 53BP1, reduced fork stalling and ultimately cell survival. Treatment with histone deacetylase inhibitors can selectively sensitise irinotecan resistant cells, thereby overcoming drug resistance.

Since TSA treatment was able to efficiently reverse multiple resistance-associated phenotypes described above, we next examined if TSA could selectively sensitise irinotecan-resistant cells and thus reverse drug resistance. An optimal TSA dose (10 or 50 nM) that does not affect survival in the absence of irinotecan treatment was selected for co-treatment clonogenic survival assays (Supplementary Figure S13). RKO and RSC316 cells were pre-treated with 10 nM TSA and irinotecan sensitivity was measured by clonogenic survival assays. We observed a stark reversal of resistance, particularly at lower irinotecan doses in resistant but not parental cells (Figure 6G). TSA furthermore sensitised multiple irinotecan-resistant cells to irinotecan (Supplementary Figure S14). Further consolidating this finding, treatment with an alternative HDAC inhibitor, Panobinostat (LBH589) also led to increased fork stalling (Figure 6H) and irinotecan sensitivity (Figure 6I). Finally, we examined whether changes in chromatin acetylation are a hallmark of tumour formation and progression in colorectal cancer. Normal human colon tissue, primary and metastatic CRC tissues were subjected to immunohistochemistry (IHC) using H4K16ac antibodies. CRC tissues displayed a significant reduction in H4K16ac staining with a progressive decline in % positive cells as the tumour advanced from grade I, II to III (Figure 7). We conclude from these experiments that alterations in the chromatin acetylation landscape are hallmarks of CRC, underlying irinotecan resistance, and subsequent perturbation using HDAC inhibitors can mechanistically reverse resistance.

## DISCUSSION

The response to the chemotherapy agent irinotecan during colorectal cancer treatment is in most cases lost due to onset of drug resistance. Mechanisms promoting resistance are poorly characterized and a clearer understanding will allow for the identification of suitable treatment alternatives. We report that the mechanism of resistance onset is not due to deregulation of TOP1 or TDP1 but due to improved DNA DSB repair as observed in multiple independently generated irinotecan-resistant clones. The increased DNA DSB repair rate was due to changes in the chromatin acetylation landscape, in particular H4K16ac, and subsequent inhibition of histone deacetylases selectively sensitized irinotecan-resistant but not parental cells.

We generated irinotecan resistant clones from two well-studied colorectal cancer cell lines, RKO and DLD1. This approach provided an invaluable control, allowing for direct comparison of changes that have occurred as a result of continuous irinotecan exposure. Such controls are invariably much more difficult to achieve when directly studying patient samples. Using these research tools, we found that TDP1 upregulation or TOP1 loss was at the very least ruled out as common mechanisms of resistance. Resistant clones accumulated similar levels of toxic DNA DSBs upon irinotecan treatment compared to the parental cell line, which further rules out differences in DNA SSB repair capacity, drug delivery/efflux, and the ability of irinotecan to cause damage as possible mechanisms of resistance. Resistant clones were still able to trigger cell cycle arrest in response to irinotecan treatment but they appeared to escape this arrest more quickly, subsequently sparing cell death by apoptosis. This difference correlated well with the increased ability of the resistant clones to clear DSBs as measured by faster rates of clearance of the canonical DSB repair factors RAD51 and pRPA (52). Cross-resistance was observed with hydroxyurea and oxaliplatin treatments ruling out hyper-elimination of drugs, as a mechanism of resistance, by drug efflux pumps for example, given that this action is likely to be specific to drug type. The similar number of irinotecan-induced DSBs and TOP1cc, measured by two independent techniques, further confirm that irinotecan is still capable of inflicting comparable damage in both parental and resistant clones.

Whilst we observed an increase in DNA DSB repair, we saw no changes in the levels of DSB repair factors and associated endonucleases, using immunoblotting and microarray profiling. We were inclined therefore to understand whether events leading up to damage recognition and repair could contribute to the onset of irinotecan resistance. A fine balance of histone modifications (methylation and acetylation) is known to affect DNA repair dynamics (54). For instance, 53BP1 is recruited to damage sites by direct interaction of its tandem Tudor repeats with histone H4K20me1 and H4K20me2 (55–57). Tip60-mediated histone H4K16 acetylation was conversely shown to reduce the binding of 53BP1 to methylated histones, thus regulating the competitive recruitment of 53BP1 and BRCA1 to DNA DSBs (53). This impacts on the extent of DNA end-resection achieved and subsequently the repair pathway utilised for DSB repair (53,58–60). In multiple resistant clones, we observed faster but transient accumulation of 53BP1 at damage sites and subsequently identified a reduction in the level of H4K16 acetylation. Treatment with the HDAC inhibitor TSA results in enrichment of H4K16Ac, re-adjusts the rate of 53BP1 accumulation, increases fork stalling and ultimately selectively sensitises irinotecan-resistant clones to irinotecan (Figure 6). In contrast, we observed no irinotecan sensitisation in the TSA treated parental RKO cell line. The lack of sensitisation in the parental cell line may be TSA dose dependent, or it may reflect differences in repair pathway choices between the RKO parental cell line and the resistant clones.

How these observed differences ultimately promote irinotecan resistance is not entirely clear. What is clear is that cross-resistance is shared with treatments that rely at least in part on the HR pathway for repair, i.e. oxaliplatin and HU. It is possible that loss of H4K16Ac in response to prolonged irinotecan treatment targets breaks normally destined for HR repair towards NHEJ by recruiting 53BP1 to the break site more readily. Such sharing of repair workload between two repair pathways would lessen the burden on the HR machinery, which is already improved as shown by rapid RAD51 foci clearance. Thus, it could provide a feedback mechanism through which the evolving cancer cells attempt to improve both DSB repair pathways to combat irinotecan induced DNA damage.

We note that HDAC inhibitor treatment will affect the acetylation status of a wide range of targets, thus it is possible that the acetylated target responsible for mediating changes in DSB repair kinetics is not exclusively restricted to H4K16 (61,62). Notably, suppression of histone deacetylation using other inhibitors of HDACs also sensitizes resistant, but not parental, cells to irinotecan. These findings are consistent with a model in which alterations in H4K16ac impact DSB repair rate and, as a result, cellular survival following irinotecan. In support of the involvement of H4K16Ac regulation as a resistance mechanism, we further show that loss of Tip60 in the parental RKO cell line reduces H4K16Ac and promotes partial resistance to irinotecan. The partial resistance achieved implies that resistance may be triggered through altered regulation of both acetylation and deacetylation dynamics. Whilst we observed stark reversal of resistance using TSA treatment, we did not observe complete reversal suggesting that other mechanisms of resistance may exist alongside deregulated H4K16Ac. Indeed, we identified two subpopulations of resistant clones derived from the RKO parental cell line, both of which share deregulation of H4K16Ac in response to irinotecan treatment. It is therefore conceivable that the more resistant population possesses additional mechanisms of resistance and that changes in H4K16ac is a novel, but not the only, mechanism of resistance. Importantly, HDAC inhibitors are currently FDA approved for clinical use with multiple agents in clinical trials, which could be tested for the targeted treatment of irinotecan-resistant CRC.

In agreement with our main findings, we were able to rule out other possible mechanisms of resistance. Whilst we observed remarkable irinotecan sensitization using PARP inhibitor treatment, we observed a similar extent of sensitization in parental cell lines. It has been reported that TOP1cc triggers replication fork reversal that is controlled by PARP1 and the RECQ1 helicase (16,17). This occurs in an effort to avoid direct collision of the replication machinery with the TOP1-PDB and therefore DSB formation, thus allowing more time for repair of TOP1-PDBs most likely via the SSB repair pathway. Whilst PARP1 depletion had mild impact on irinotecan sensitivity, it did not restore sensitivity of irinotecan-resistant clones, suggesting that PARP1-regulated fork reversal is unlikely to contribute to resistance. In support of this notion, there was no difference in the initial numbers of DSBs following irinotecan treatment. Interestingly, PARP1 is known to promote TDP1-dependent repair of trapped TOP1-cc in preparation for fork restart (12,14). Our data further argue against a role for TDP1/PARP1 axis as a mechanism of resistance since TDP1 depletion was similarly not able to reverse resistance. In contrast to PARP1 depletion, poisoning the HR machinery by PARP inhibition sensitized both parental and irinotecan resistant CRC cells to irinotecan, which is consistent with the published literature in other cancer types (63). Thus, PARP inhibition interferes with the normal processing of irinotecan-mediated damage but deregulated PARP activity is unlikely to promote irinotecan resistance.

These findings have important clinical implications that can now be explored. For instance, a clinical study examining the efficacy of PARP or HDAC inhibitors in both irinotecan responsive and non-responsive patients should be of major interest, particularly since these inhibitors are already FDA-approved. Interestingly and in support of our findings, it has been reported that loss of monoacetylated forms of histone H4 appears early and accumulates during the tumorigenic process in skin cancer (64). The losses occurred predominantly at the acetylated Lys16 of histone H4. Here, we also report that changes in chromatin acetylation in patient derived tissues are a hallmark of colorectal cancer initiation and progression (Figure 7). It will now be interesting to examine if loss of H4K16ac is a hallmark of irinotecan resistance in CRC tissues, using xenograft models or clinical samples, in follow-up studies. Finally, our findings suggest that irinotecan resistant CRC should not be treated alternatively with an oxaliplatin-based regime (such as FOLFOX) since cross-resistance is apparent. However, they are unlikely to generate additional resistance to 5-FU and radiation therapies since cross-resistance was not observed.

In summary, we identify changes in histone acetylation in irinotecan resistant colorectal cancer and further reveal inhibitors of HDACs as promising means to overcome resistance. We suggest that more attention should be given to epigenetic changes such as acetylation when considering resistance to TOP1 targeting therapies.

## Accession Number

Microarray data are available in the ArrayExpress database (www.ebi.ac.uk/arrayexpress) under accession number E-MTAB-5184.

## Supplementary Material

Supplementary Data
