# Supplementary material for: Epigenetic changes in histone acetylation underpin resistance to the topoisomerase I inhibitor irinotecan
Source: Nucleic Acids Res. 2016 Oct 26;45(3):1159–76. doi: 10.1093/nar/gkw1026 (PMC5388393; doi:10.1093/nar/gkw1026)
Supplement: Supplementary Data [file gkw1026_Supp.zip › nar-01653-d-2016-File009.pdf]

Supplementary Figure 1

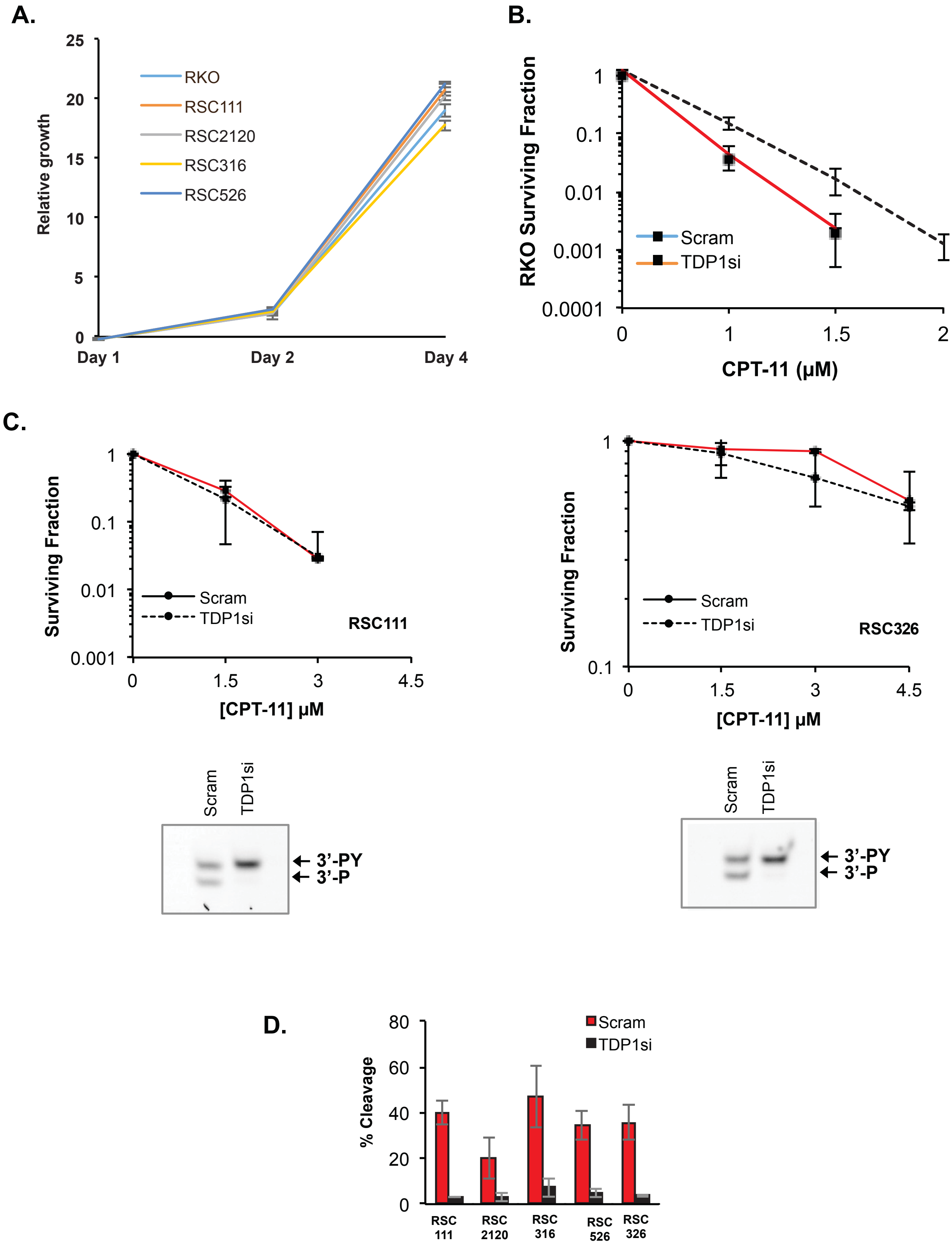

Supplementary Figure 1. TDP1 depletion does not restore irinotecan sensitivity in irinotecan resistant colorectal cancer cell lines. (**A**) 2000 cells per indicated cell line were seeded in to wells of a 96-well dish. Titreblue readings were obtained on Day 1, day 2 and Day 4. All readings were normalised to that obtained on Day 1. Relative growth for one representative biological replicate ± STD is shown. **(B, C)** A clonogenic survival assay was conducted following treatment with the indicated CPT11 doses on TDP1-depleted RKO (C) and RSC111 and RSC326 cells (B, upper panel). Data for 3 independent experiments ± STD is shown. WCE prepared from the corresponding TDP1-depleted cells were subjected to a TDP1 activity assay and the reaction products separated by 20% denaturing PAGE (B, lower panels). The substrate (3'PY) and product (3'P) are indicated for one representative experiment of three. **(D)** Quantification of the product of TDP1 catalytic activity showing % cleavage of the substrate (3'PY) to product (3'P) from 3 biological replicates. Data are the average ± STD.

Supplementary Figure 2

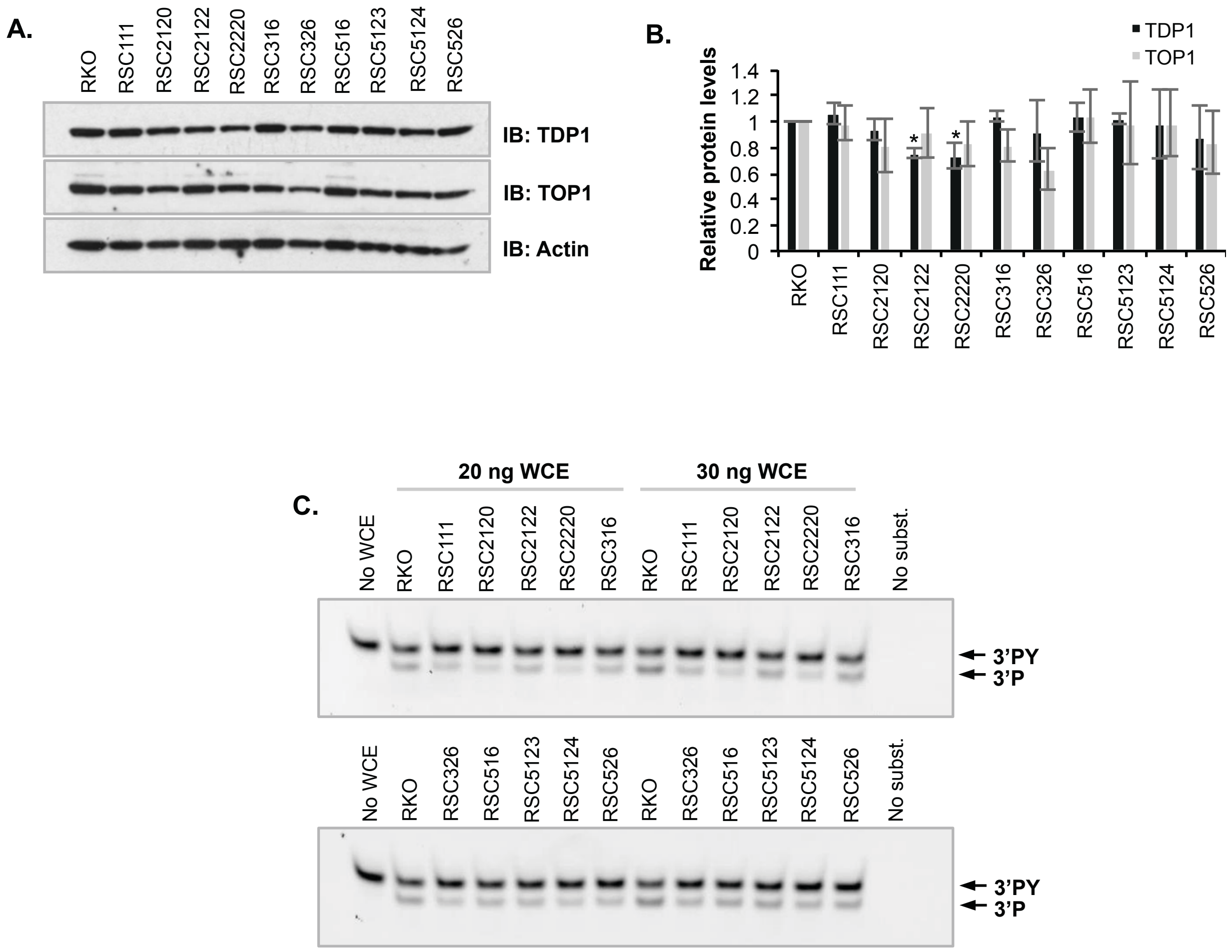

Supplementary Figure 2. TDP1 and TOP1 protein levels and activity are not significantly altered in the irinotecan resistant clones (A) Whole cell extract prepared from parental RKO and irinotecan-resistant RKO derived clones were separated by 10% SDS-PAGE and immunoblotted using anti TDP1 (ab4166; Abcam) , TOP1 (SC-32736; Santa Cruz Biotechnologies) , or actin (A4700; Sigma-Aldrich) antibodies. **(B)** TDP1 and TOP1 bands were quantified using ImageJ software, normalised to actin and then expressed as fold change relative to expressions in the parental RKO cells. Data are the average of three independent biological replicates ± STD. **(C)** Whole cell extract prepared from parental RKO and resistant RKO derived clones were incubated with oligonucleotides harbouring a 3'-phosphotyrosine modification (3'PY) and 5'-fluorophore to monitor TDP1 catalytic activity. Reaction products were separated by 20 % denaturing PAGE and the gel imaged using a FujiFilm Fluor Imager FLA-5100 at 635 nm. Arrowheads denote the position of substrate (3'PY) and product (3'P).

Supplementary Figure 3

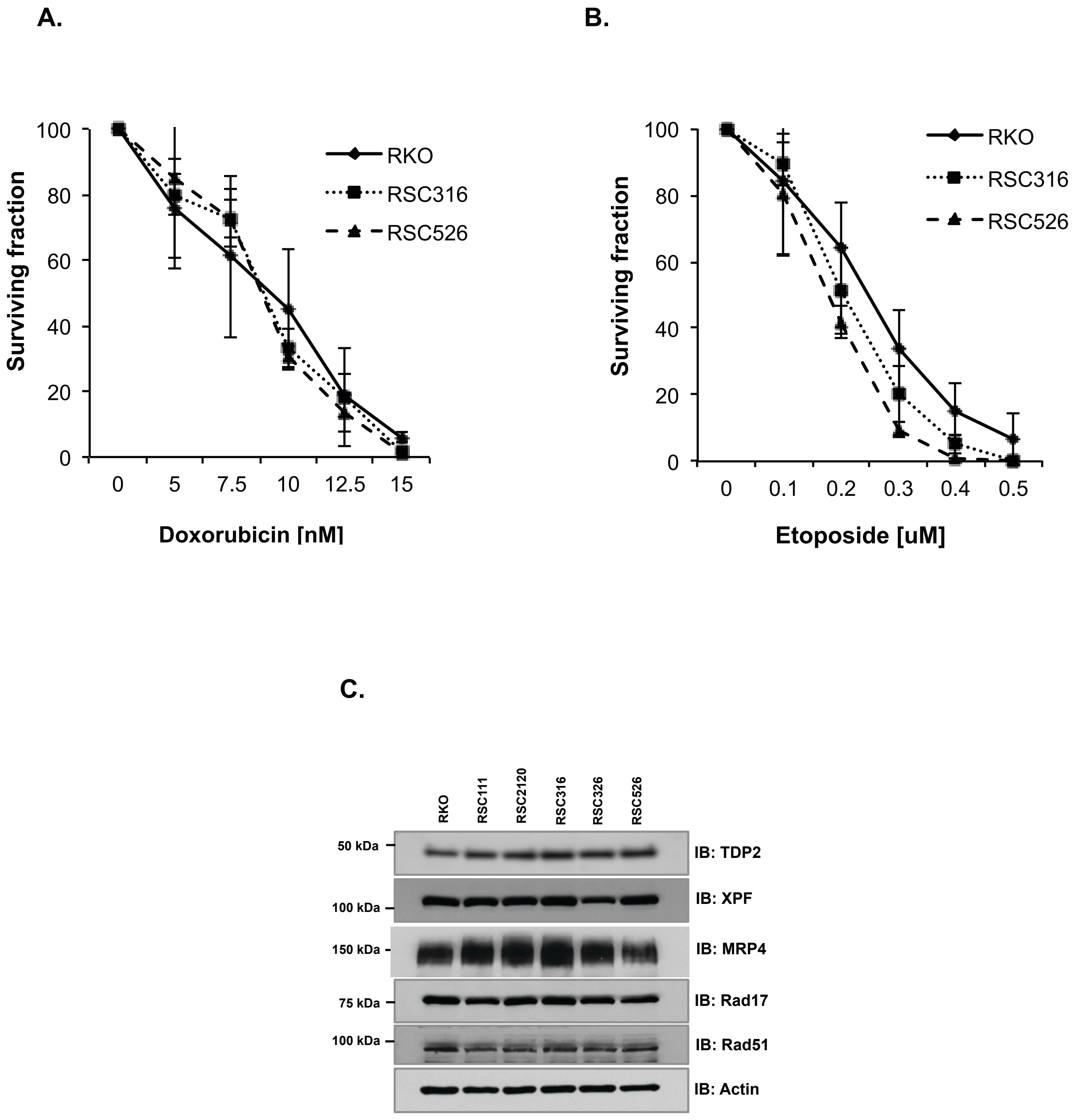

Supplementary Figure 3. Irinotecan-resistant cells do not diplay cross-resistance with TOP2 targetting drugs. Parental and irinotecan resistant cells were treated with the indicted doses of doxorubicin (A) or etoposide (B) and sensitivity calculated using clonogenic survival assays. (C) Cell lystaes were fractionated by SDS PAGE analysed by immunoblotting using antibodies for TDP2, XPF, MRP4, Rad17, and Rad51 and actin.

Supplementary Figure 4

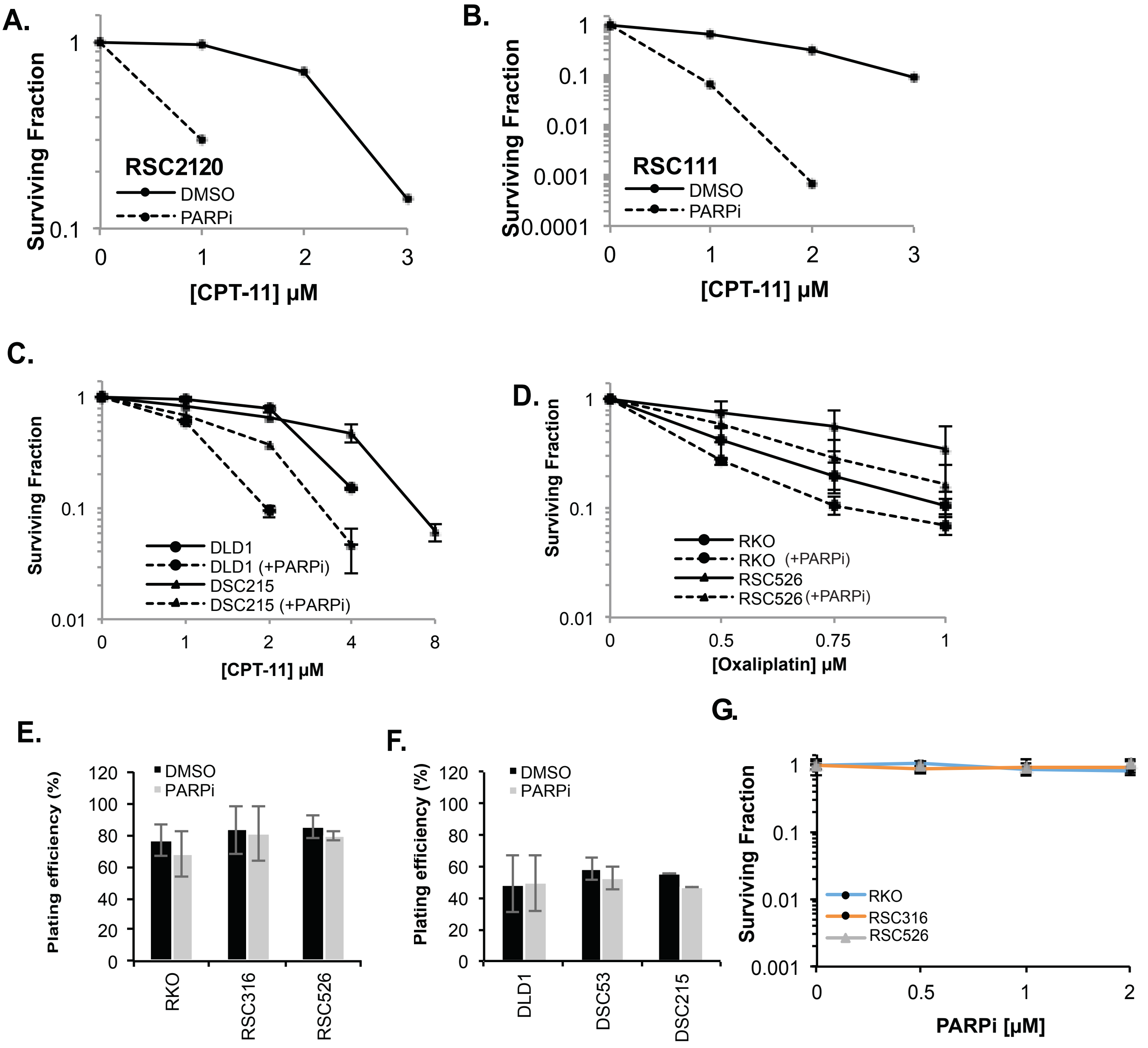

Supplementary Figure 4. The PARP inhibitor olaparib non-mechanistically reverses irinotecan resistance but not oxaliplatin cross-resistance. The RSC2120 **(A)**, RSC111 **(B)** and the DLD1 and derived DSC215 resistant cells **(C)** were incubated with 1 µM olaparib and indicated CPT-11 doses for the duration of colony formation. Surviving fraction was calculated as the surviving colony fraction^{treated}/surviving colony fraction^{untreated} where surviving colony fraction = colonies counted/total cells seeded. The graphical representation shows the average surviving fraction for three independent experiments ± STD. **(D)** Parental RKO and derived resistant RSC526 cells were mock treated or incubated with 1 µM PARP inhibitor olaparib, followed by indicated oxaliplatin treatment. Colonies were fixed, stained and counted and the surviving fraction calculated. Data are the average of three biological replicates ± STD. Plating efficiencies in CPT-11 untreated cells for mock or PARPi treated RKO, RSC316 and RSC526 are shown in **(E)** and for DLD1, DSC53 and DSC215 in **(F)**. Plating efficiencies were calculated as (colonies obtained/number of cells seeded) and results for 3 independent repeats ± STD is shown. (**G**) Cells were exposed to the indicated concentrations of olaparib and survival calculated from two independent experiments ± range.

Supplementary Figure 5

A.

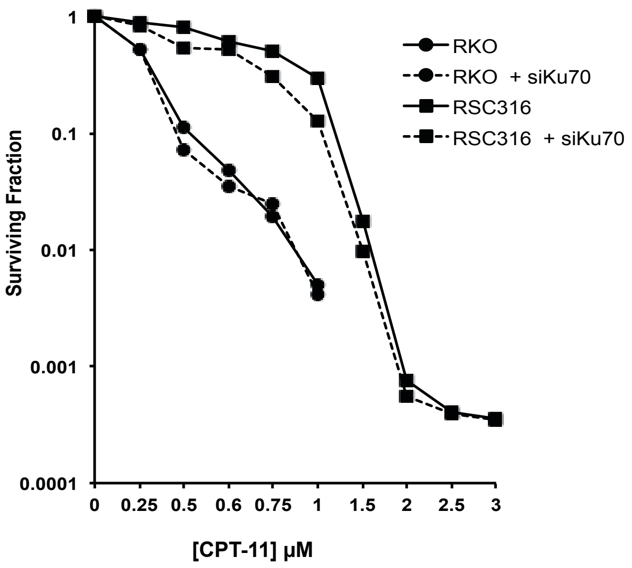

B.

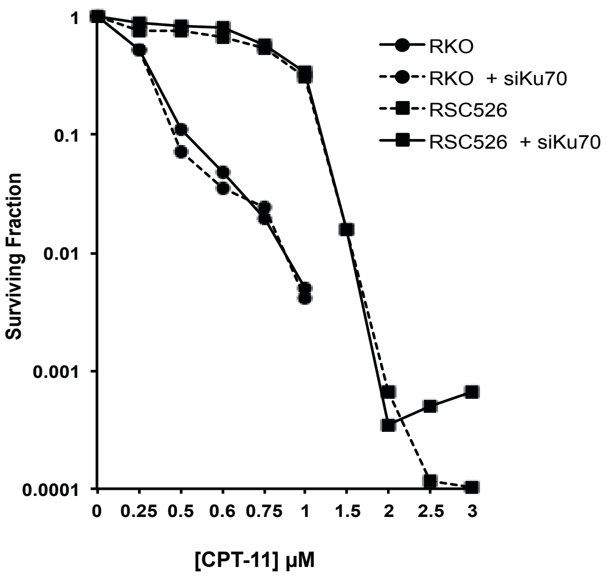

C.

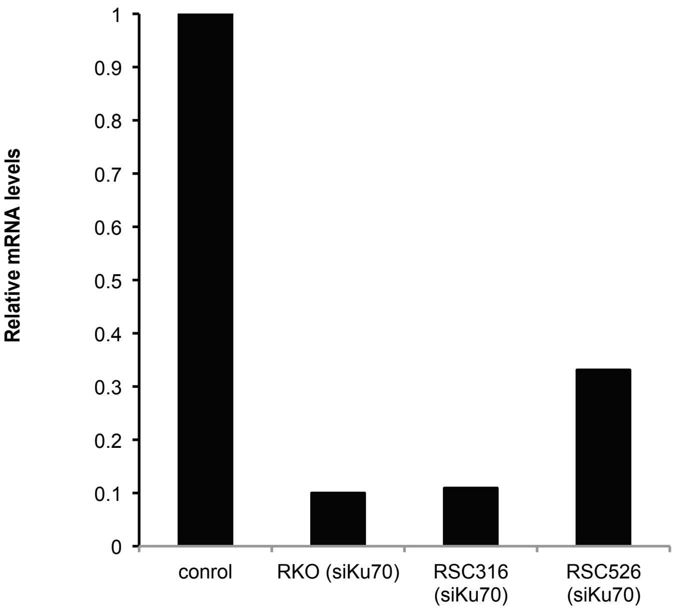

Supplemetary figure 5. Depeltion of KU70 does not overcome irinotecan resistance in CRC. (A,B) Survival assays for cells treated with scrambled siRNA or siRNA against KU70 and exposed to the indicated concetrations of CPT-11. (C) KU70 mRNA levels were examined by qPCR.

Supplementary Figure 6:

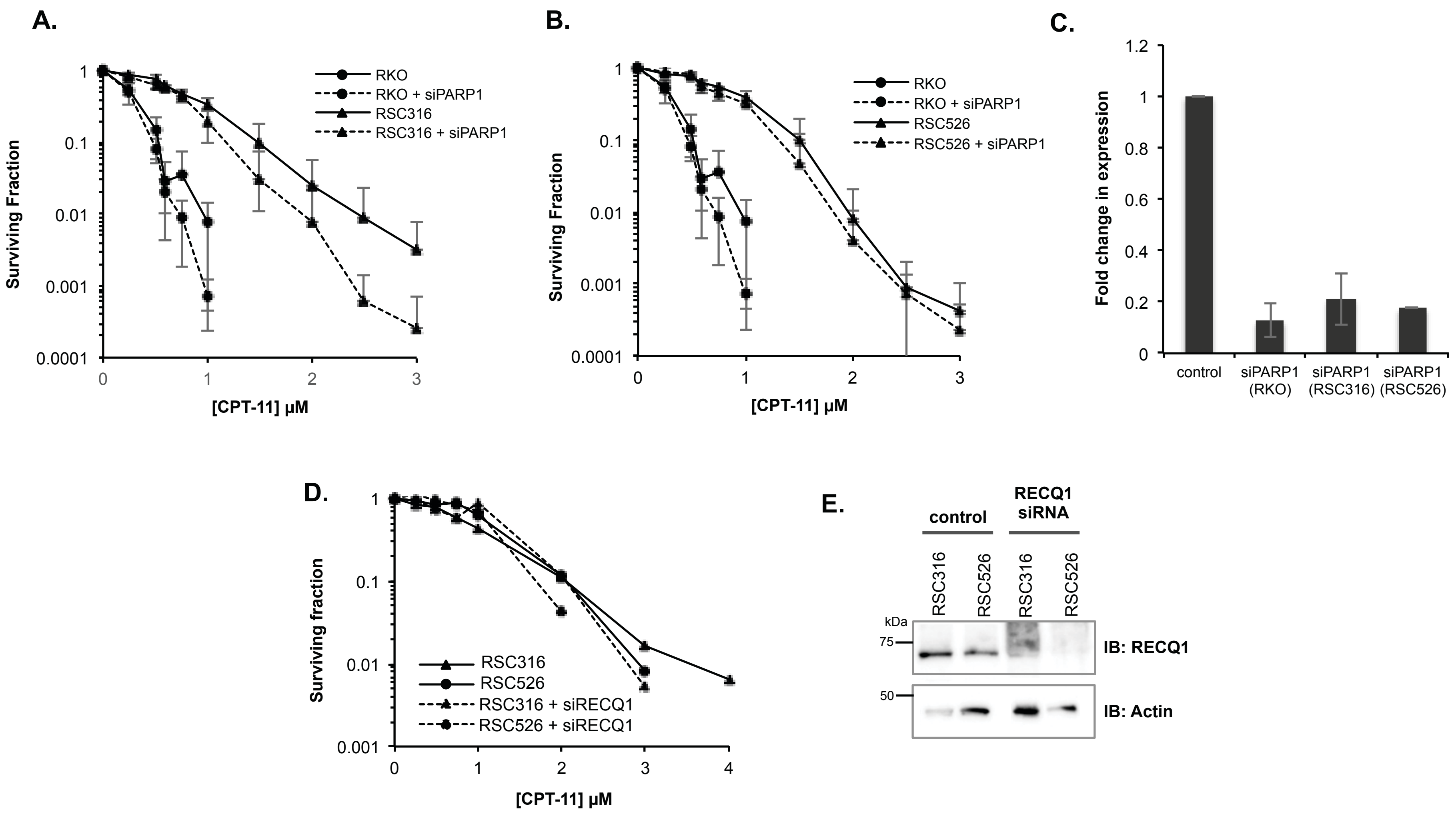

Supplementary Figure 6. Depletion of PARP1 or RECQ1 helicase does not restore irinotecan sensitivity in irinotecan-resistant colorectal cancer cells. Parental CRC cells (RKO), irinotecan-resistant clones RSC316 **(A)** and RSC526 **(B)** were treated with PARP1 siRNA and then exposed to the indicated concentrations of CPT-11 for the duration of colony formation (7-12 days). The surviving fraction was calculated as the surviving colony fraction^{treated} / surviving colony fraction^{untreated}, where surviving colony fraction = colonies counted / total cells seeded. Data are the average of 3 biological replicates ± STD. **(C)** mRNA transcript levels were quantified by quantitative PCR and expressed as fold change to mRNA levels in RKO cells. Results are the average of 2 biological replicates ±SEM. **(D)** RSC316 and RSC526 cells were treated with RECQ1 siRNA and then exposed to the indicated concentrations of CPT-11 for the duration of colony formation. Survival was calculated as above and data from a representative experiment is shown. **(E)** Lysate of cells untreated or treated with RECQ1 siRNA were fractionated on SDS PAGE and immunoblotted with anti RECQ1 (H-110 Santa Cruz) or anti-actin (AC-15 Abcam) antibodies.

Supplementary Figure 7

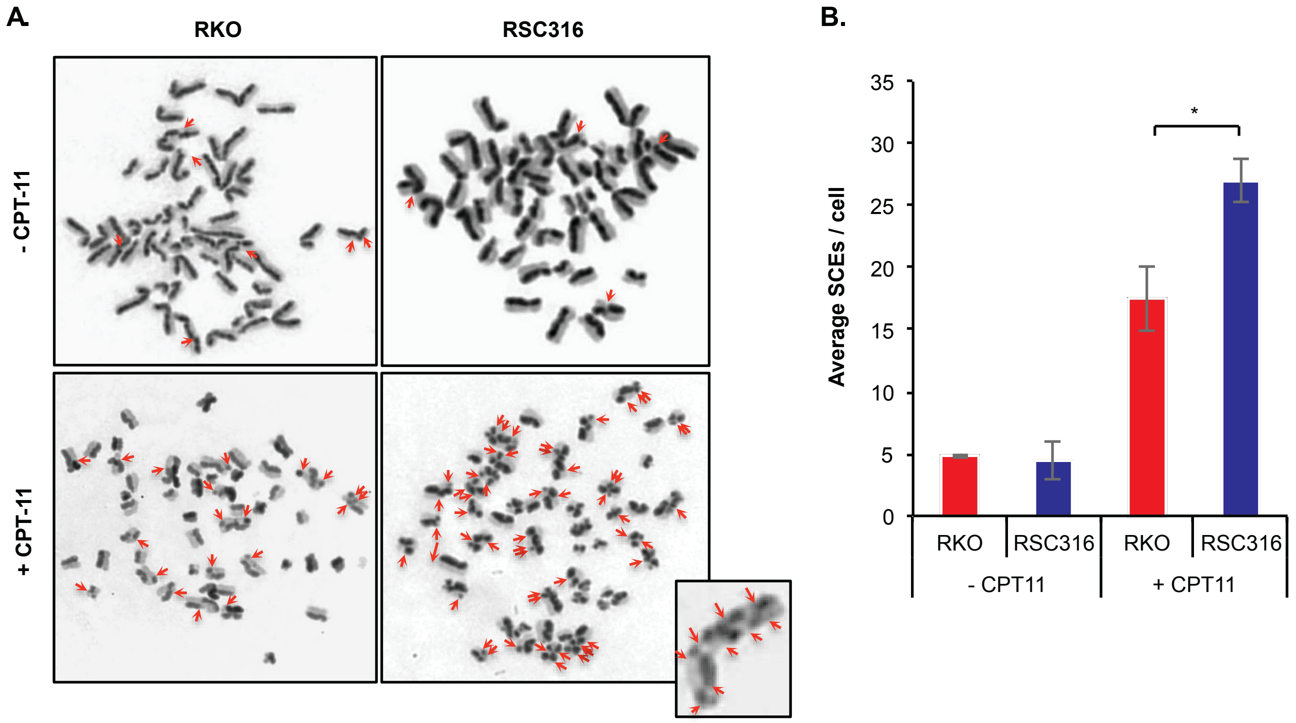

Supplemetary Figure 7. Irinotecan-resistant cells display more SCEs than parentals. (A) Exponentially growing parental RKO and its irinotecan-resistant RSC316 cells were incubated with 20 μM 5-bromodeoxyruridine for two cell generations (~ 45 hr), mock treated with DMSO “-CPT-11” or with 1 μM CPT-11 “CPT-11” for 1 hr, and metaphase spreads were incubated with Hoechst 33258, exposed to 355nm UV light for 1 hr, and stained with 3% Giemsa. A representative field showing SCEs in RKO and RSC316 cells is shown. (**B**)The average number of SCEs per cell ± mean was quantified from at least 40 cells from 2 biological repeats.

Supplementary Figure 8:

A.

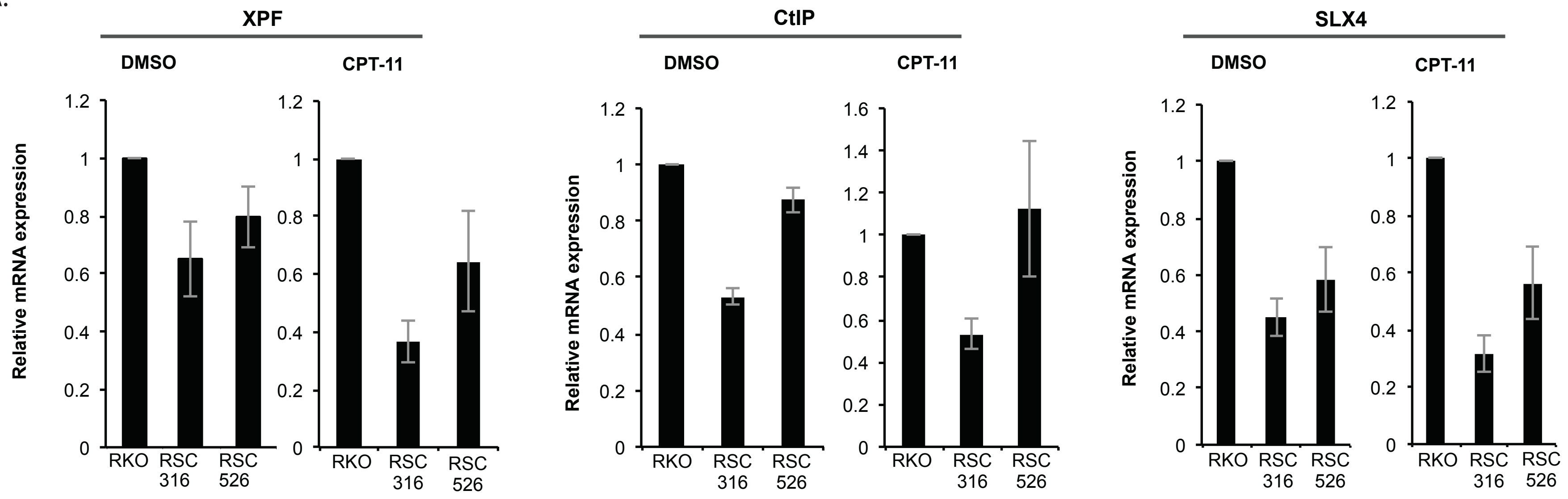

B.

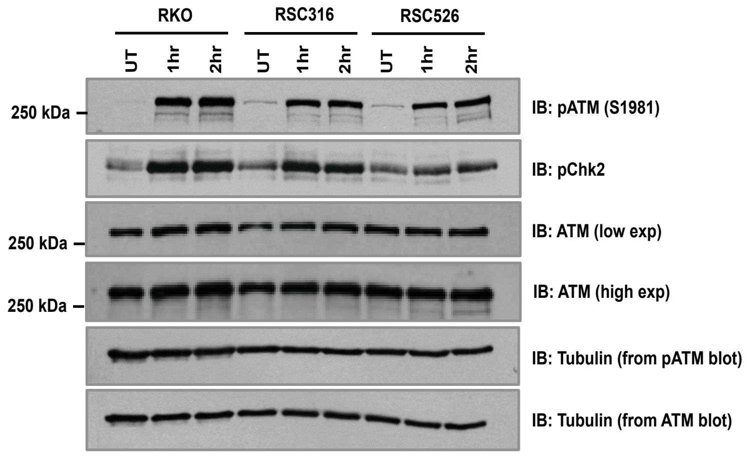

Supplemetary Figure 8. Upregulation of HR repair nucleases or ATM signalling does not account for irinotecan-resistance. (A) mRNA transcript levels were quantified in the indicated cells using quantitative PCR. Data were normalised to expression level in parental RKO cells and presented as relative mRNA expression. Results are from 2 biological replicates ± SEM. **(B)** The indicated cells were treated with 1μM SN38 and cell lysates fractionated by SDS PAGE and immunoblotted using anti-ATM and anti-pATM antibodies.

Supplementary Figure 9

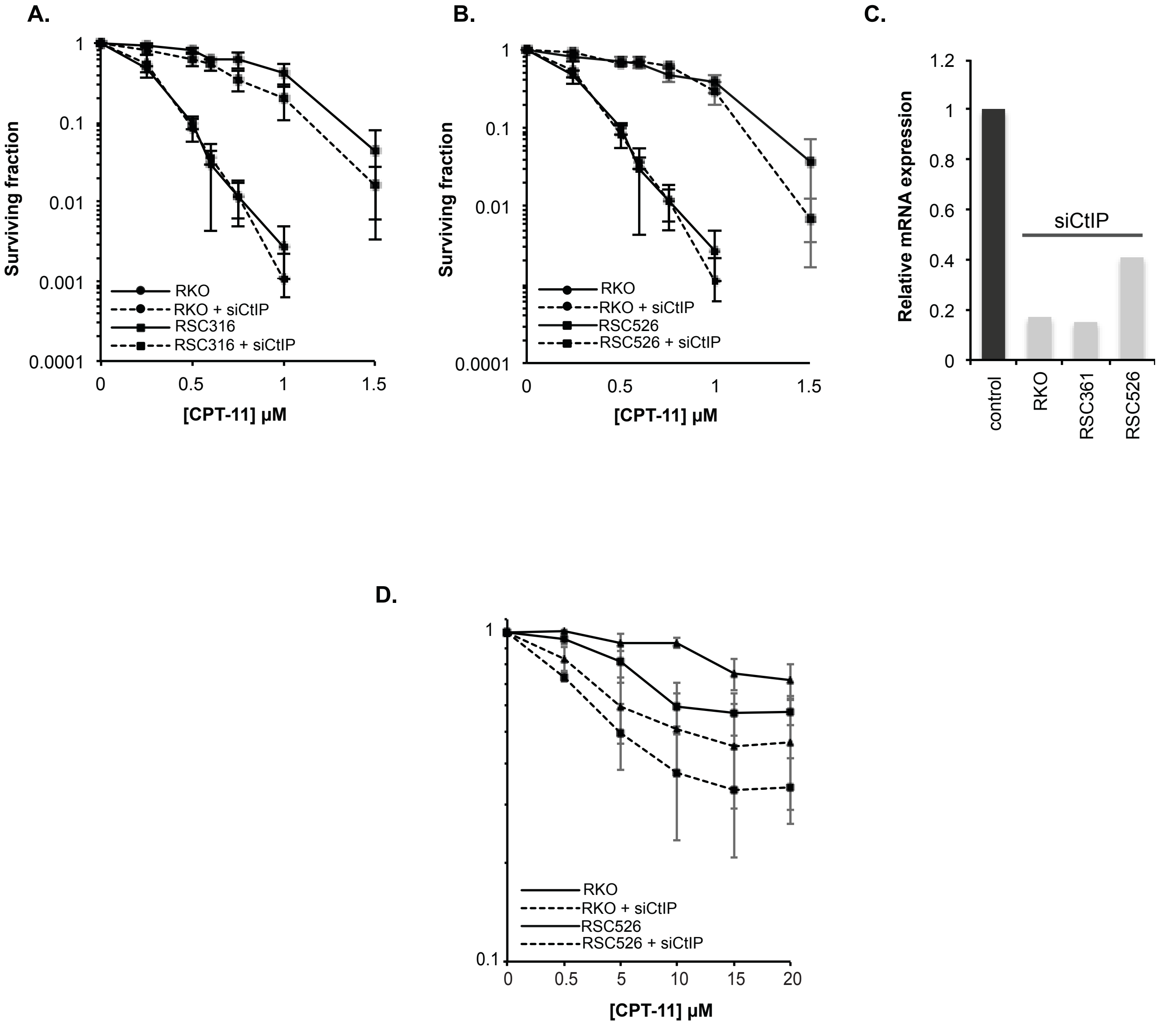

Supplementary Figure 9. CtIP depletion does not selectively overcome irinotecan resistance. RKO parental and the irinotecan-resistant RSC316 (**A**) and RSC526 (**B**) cells were treated with a scrambled siRNA or siRNA against CtIP, followed by exposure to the indicated concentrations of CPT-11 ***for the duration of colony formation***. Survival was calculated from 3 biological replicates ± STD. **(C)** mRNA transcript levels were quantified from the indicated cell lines using quantitative PCR. Data is a representation of one experiment. **(D)** RKO and resistant cell lines were treated with scrambled siRNA or siRNA against CtIP, followed by exposure to the indictaed concentrations of CPT-11 ***for 1 hr***. Survival was calculated from 3 biological replicates ± STD.

Supplementary Figure 10:

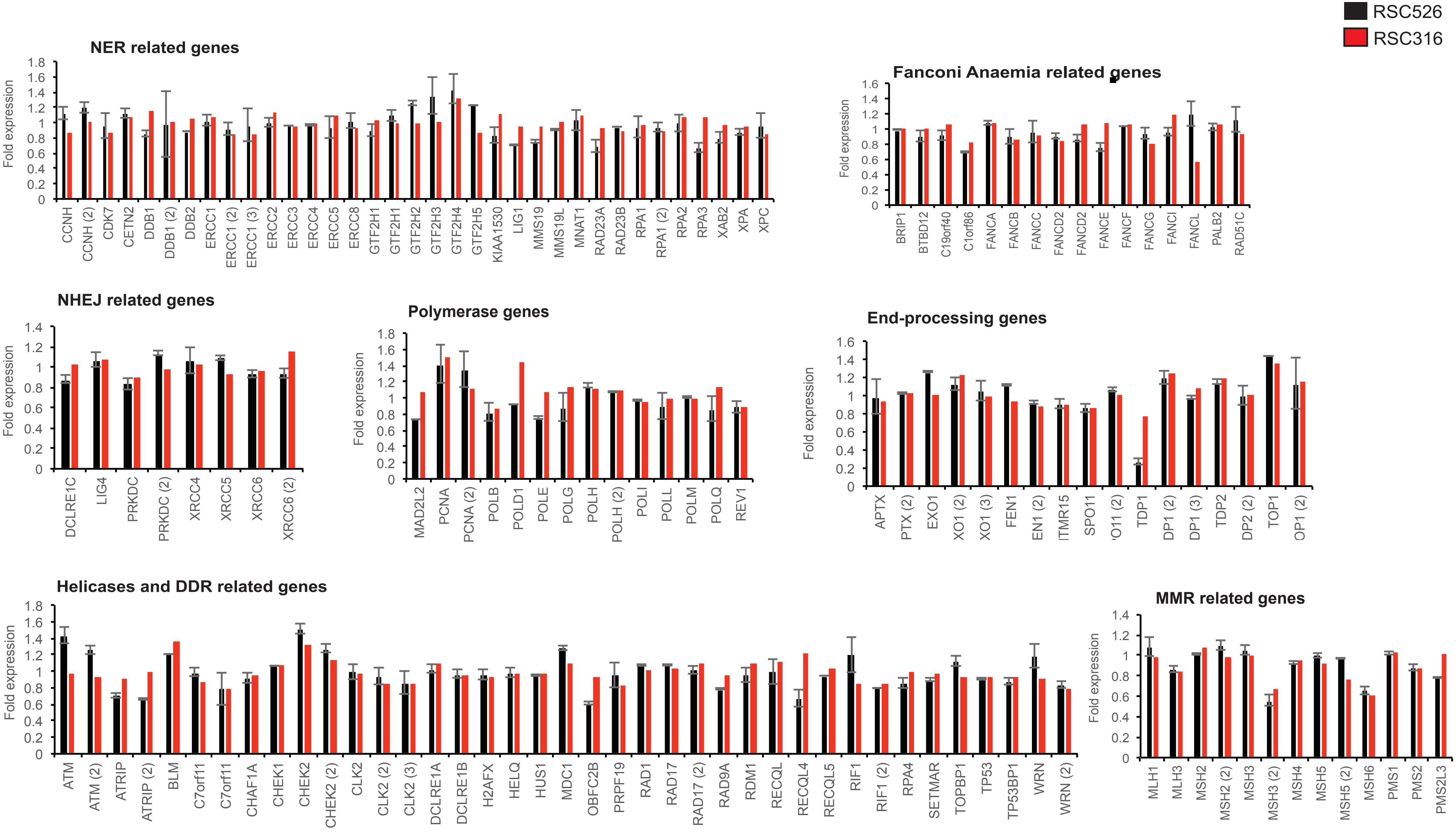

Supplemetary Figure 10. mRNA profiling of genes predicted to participate in irinotecan resistance. Transcript levels of the indicated cohort of genes was quantified by microarray analyses in parental RKO and the indicated irinotecan resistant cells, following treatment with irinotecan. Expression level was normalised to RKO and presented as fold change +/- range. Results are averages of two independent experiments.

Supplementary Figure 11

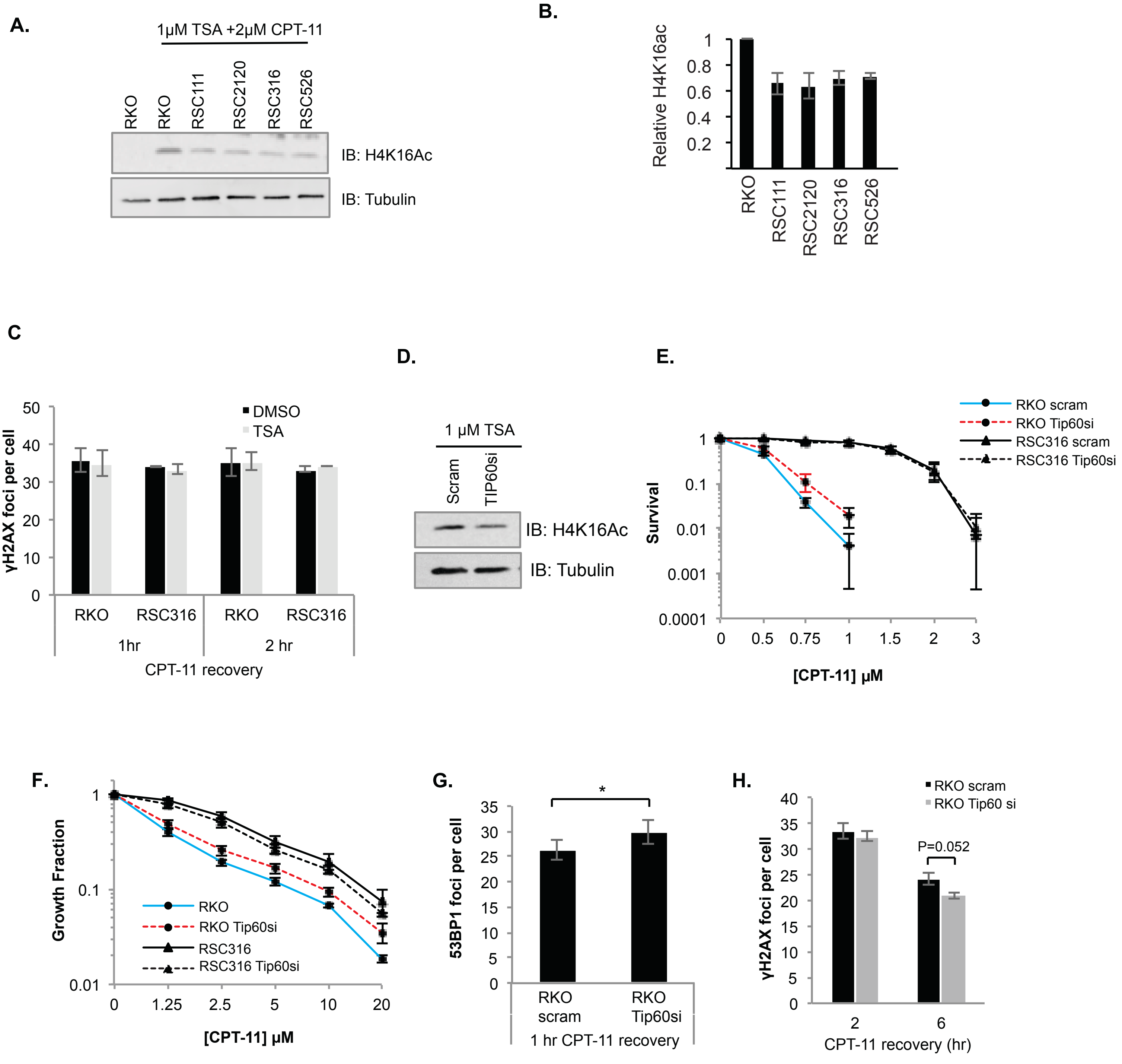

Supplementary Figure 11. Tip60 depletion echos irinotecan resistance phenotype. (A,B) The indicated cell lines were treated with TSA and CPT-11 and analysed by immunoblotting. (**C**) RKO and RSC316 cells were pre-treated with 1 μM TSA for 2 hr, followed by treatment with 2 μM CPT-11 for 1.5 hr and indicated recovery times of 1 and 2 hr. Cells were fixed, permeabilised and analysed by immunostaining for γH2AX. The number of foci per cell for 36 cells was counted manually on a Nikon Eclipse e-400 microscope and the average number of foci per cell for three independent experiments is shown ± STD. Cells were treated with a scrambled siRNA or siRNA against Tip60, followed by WB (**D**) or exposure to the indicated concentrations of CPT-11 for an irinotecan survival assay (**E**) and a Cell-Titre Blue viability assay **(F)**. Surviving and growth fractions were calculated from 3 biological replicates ± STD. **(G)** Tip60 depleted RKO cells were treated with 2 μM CPT-11 for 1.5 hr and left to recover for 1 hr. Cells were fixed, permeabilised and immunostained with 53BP1 antibodies. 53BP1 foci per cell was counted as above and data shown represents three independent repeats ± SEM. (**H**) Tip60 depleted RKO cells were treated with 2 μM CPT-11 for 1.5 hr and left to recover for 2 and 6 hr. Cells were fixed, permeabilised and immunostained with γH2AX antibodies. γH2AX foci per cell was counted as above and data shown represents three independent repeats ± STD. Asterisks denote statistical significance; * $p<0.05$; student t-test.

Supplementary Figure 12

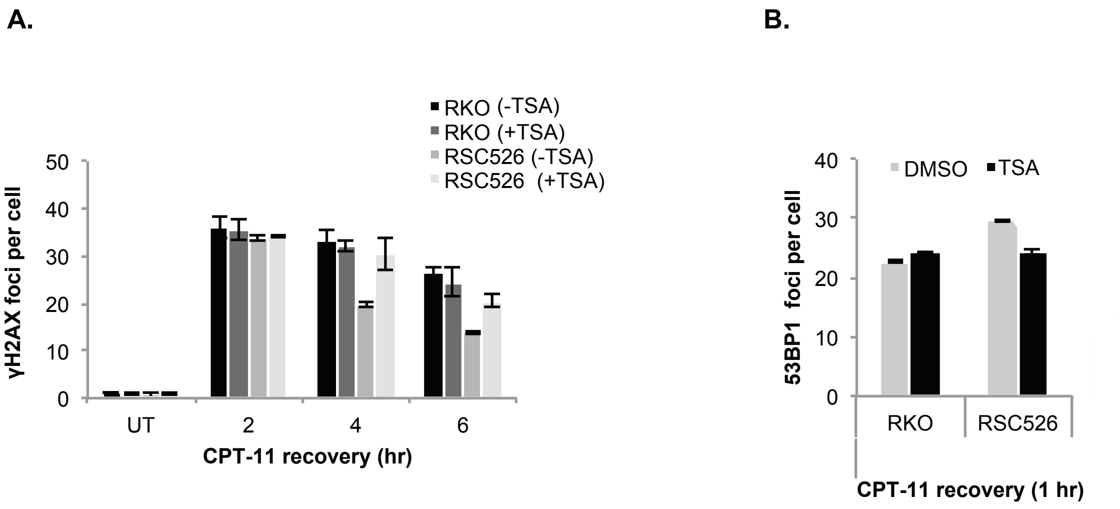

Supplementary Figure 12. TSA reverses the fast DSB repair and higher 53BP1 accumulation in the CPT-11 resistant RSC526 cells. RKO and RSC526 cells were pre-treated with 1 μM TSA for 2 hr, followed by treatment with 2 μM CPT-11 for 1.5 hr and recovery for the indicated times. Cells were fixed, permeabilised and analysed by immunostaining for γH2AX (**A**) and 53BP1 (**B**). The number of foci per cell for 50 cells was counted on a Nikon Eclipse e-400 microscope and the average number of foci per cell for two independent experiments is shown ± range.

Supplementary Figure 13

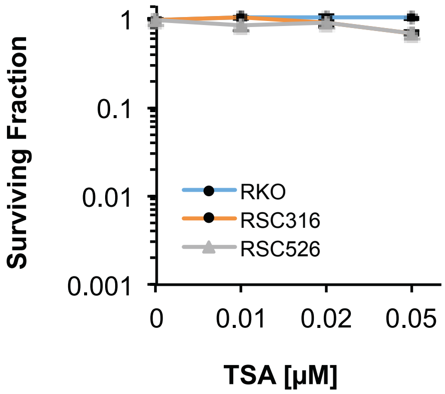

Supplementary Figure 13. Comparable sensitivity of parental and CPT-11 resistant cells to TSA alone. Cells were exposed to the indicated concentrations of TSA and survival calculated using clonogenic assays. Data are the average of two independent experiments +/- range.

Supplementary Figure 14

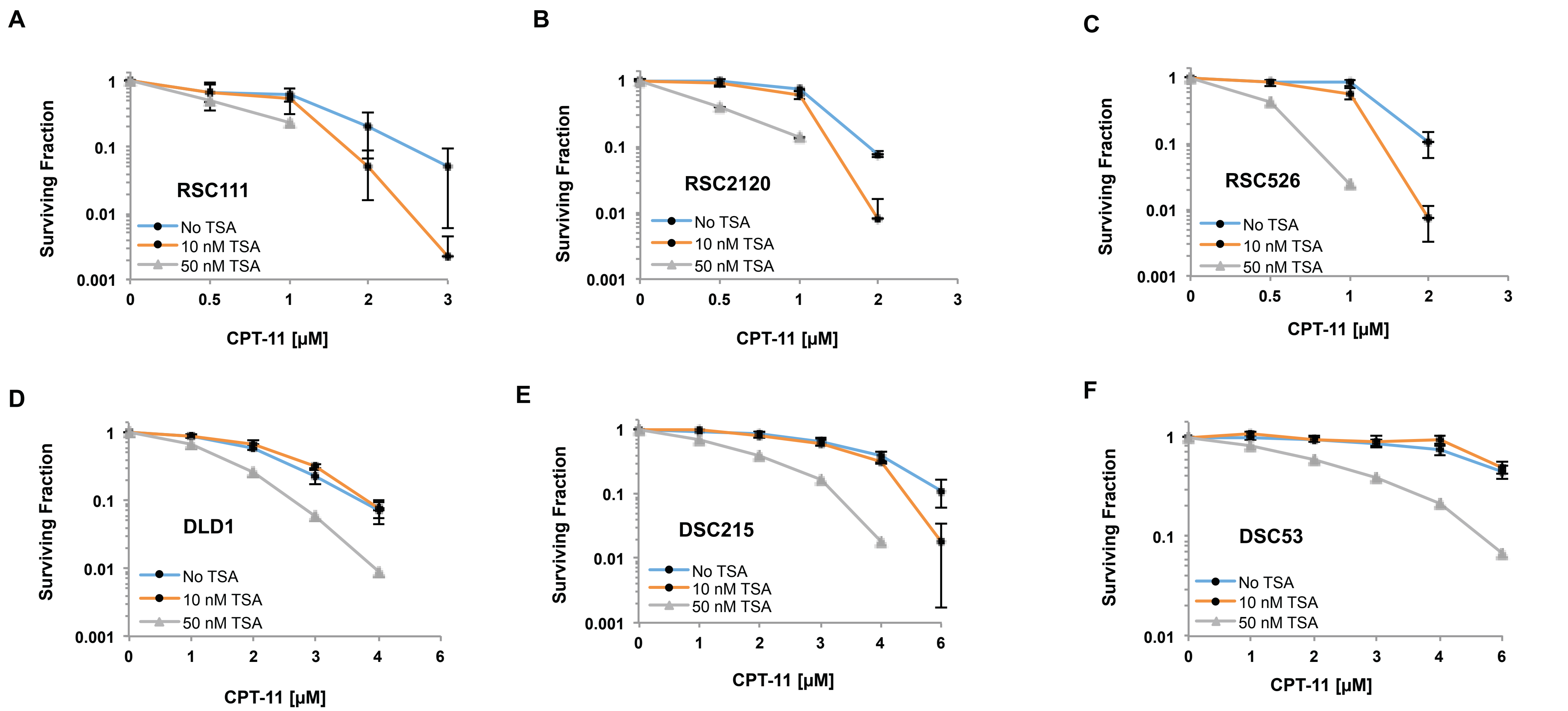

Supplementary figure 14. Trichostatin A treatment sensitises multiple RKO and DLD1 derived irinotecan resistant clones to irinotecan. RSC111 (A), RSC2120 (B) and RSC526 (C), DLD1 (D), DSC215 (E) and DSC53 (F) cells were pre-treated with 10 nM or 50 nM TSA for 1 hr, followed by exposure to the indicated concentrations of CPT-11 for the duration of colony formation. Survival fraction was calculated from 3 biological replicates ± SEM.
