# Supplementary figures and images for: Epigenetic changes in histone acetylation underpin resistance to the topoisomerase I inhibitor irinotecan
Source: Nucleic Acids Res. 2016 Oct 26;45(3):1159–76. doi: 10.1093/nar/gkw1026 (PMC5388393; doi:10.1093/nar/gkw1026)

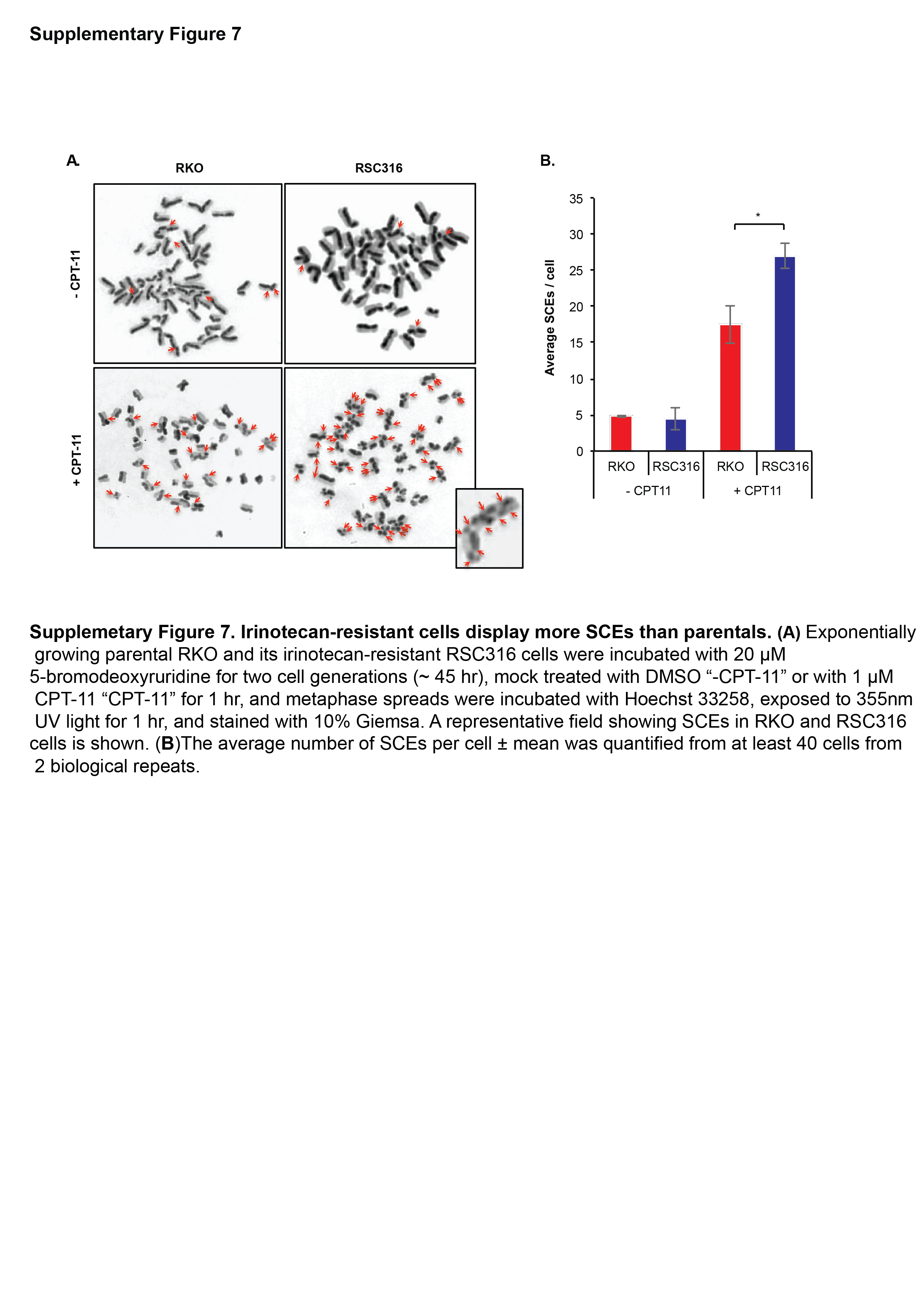

Supplement: Supplementary Data [file gkw1026_Supp.zip › CRCR_Suppl_Figure_7-01.png]
